# Post-Stroke Inhibition of Induced NADPH Oxidase Type 4 Prevents Oxidative Stress and Neurodegeneration

**DOI:** 10.1371/journal.pbio.1000479

**Published:** 2010-09-21

**Authors:** Christoph Kleinschnitz, Henrike Grund, Kirstin Wingler, Melanie E. Armitage, Emma Jones, Manish Mittal, David Barit, Tobias Schwarz, Christian Geis, Peter Kraft, Konstanze Barthel, Michael K. Schuhmann, Alexander M. Herrmann, Sven G. Meuth, Guido Stoll, Sabine Meurer, Anja Schrewe, Lore Becker, Valérie Gailus-Durner, Helmut Fuchs, Thomas Klopstock, Martin Hrabé de Angelis, Karin Jandeleit-Dahm, Ajay M. Shah, Norbert Weissmann, Harald H. H. W. Schmidt

**Affiliations:** 1 Neurologische Klinik und Poliklinik, Universität Würzburg, Würzburg, Germany; 2 Rudolf-Buchheim-Institut für Pharmakologie & Medizinische Klinik, Justus-Liebig-Universität, Gießen, Germany; 3 Department of Pharmacology and Centre for Vascular Health, Monash University, Melbourne, Australia; 4 Department of Pharmacology and Toxicology and Cardiovascular Research Institute Maastricht (CARIM), Maastricht University, The Netherlands; 5 National Stroke Research Institute, Florey Neuroscience Institutes, Melbourne, Australia; 6 Baker IDI Heart and Diabetes Institute, Juvenile Diabetes Research Foundation (JDRF) International Center for Diabetic Complications Research, Melbourne, Australia; 7 Abteilung Neurologie, Georg-August Universität Göttingen, Göttingen, Germany; 8 Universitätsklinik Münster, Klinik und Poliklinik für Neurologie—Entzündliche Erkrankungen des Nervensystems und Neuroonkologie, Münster, Germany; 9 Institute of Experimental Genetics, Helmholtz Zentrum München, German Research Center for Environmental Health, München, Germany; 10 Friedrich-Baur-Institut an der Neurologischen Klinik, Klinikum der Ludwig-Maximilians-Universität München, München, Germany; 11 Lehrstuhl für Experimentelle Genetik, Technische Universität München, Freising-Weihenstephan, Germany; 12 King's College London School of Medicine, The James Black Centre, Cardiovascular Division, London, United Kingdom; University of Edinburgh, United Kingdom

## Abstract

Ischemic stroke is the second leading cause of death worldwide. Only one moderately effective therapy exists, albeit with contraindications that exclude 90% of the patients. This medical need contrasts with a high failure rate of more than 1,000 pre-clinical drug candidates for stroke therapies. Thus, there is a need for translatable mechanisms of neuroprotection and more rigid thresholds of relevance in pre-clinical stroke models. One such candidate mechanism is oxidative stress. However, antioxidant approaches have failed in clinical trials, and the significant sources of oxidative stress in stroke are unknown. We here identify NADPH oxidase type 4 (NOX4) as a major source of oxidative stress and an effective therapeutic target in acute stroke. Upon ischemia, NOX4 was induced in human and mouse brain. Mice deficient in NOX4 (*Nox4* ^−/−^) of either sex, but not those deficient for NOX1 or NOX2, were largely protected from oxidative stress, blood-brain-barrier leakage, and neuronal apoptosis, after both transient and permanent cerebral ischemia. This effect was independent of age, as elderly mice were equally protected. Restoration of oxidative stress reversed the stroke-protective phenotype in *Nox4* ^−/−^ mice. Application of the only validated low-molecular-weight pharmacological NADPH oxidase inhibitor, VAS2870, several hours after ischemia was as protective as deleting NOX4. The extent of neuroprotection was exceptional, resulting in significantly improved long-term neurological functions and reduced mortality. NOX4 therefore represents a major source of oxidative stress and novel class of drug target for stroke therapy.

## Abstract 2

The identification of NOX4 as a major source of oxidative stress in stroke and demonstration of dramatic protection after stroke in mice by NOX4 deletion or NOX inhibition, opens up new avenues for treatment.

## Author Summary

Stroke is the second leading cause of death worldwide. Today, only one approved therapy exists—a drug that breaks down blood clots—the effectiveness of which is moderate, and it can only be used in about 10% of patients because of contraindications. New therapeutic strategies that are translatable to humans and more rigid thresholds of relevance in pre-clinical stroke models are needed. One candidate mechanism is oxidative stress, which is the damage caused by reactive oxygen species (ROS). Antioxidant approaches that specifically target ROS have thus far failed in clinical trials. For a more effective approach, we focus here on targeting ROS at its source by investigating an enzyme involved in generating ROS, known as NADPH oxidase type 4, or NOX4. We found that NOX4 causes oxidative stress and death of nerve cells after a stroke. Deletion of the NOX4-coding gene in mice, as well as inhibiting the ROS-generating activity of NOX with a pharmacological inhibitor, reduces brain damage and improves neurological function, even when given hours after a stroke. Importantly, neuroprotection was preserved in old male and female *Nox4* ^−/−^ mice as well as in *Nox4* ^−/−^ mice subjected to permanent ischemia. NOX4 thus represents a most promising new therapeutic target for reducing oxidative stress in general, and in brain injury due to stroke in particular.

## Introduction

Ischemic stroke has outstanding medical relevance as it is the second leading cause of death in industrialized countries [1]. Due to the aging of the population, the incidence of stroke is projected to rise even further in the future [2]. Despite tremendous research activity, with more than 100 clinical trials in human stroke patients [3], only one therapy approved by the United States Food and Drug Administration is available, i.e., thrombolysis using recombinant tissue plasminogen activator (rt-PA). However, the efficacy of rt-PA on functional outcomes is moderate at best, and more than 90% of all stroke patients must be excluded from rt-PA treatment because of over 25 labeled contraindications. Therefore, there is an unmet need for more effective therapies in acute stroke.

Although a plethora of drugs for the treatment of acute stroke are effective in animal models, their translation into clinical practice has completely failed [3],[4]. As a result, many pharmaceutical companies have withdrawn from drug discovery in this area. To overcome this lack of clinically effective neuroprotective drugs, innovative strategies are urgently needed to identify pathways that can be targeted with innovative therapies [5]. Higher quality study designs are also required [6],[7].

One such high-potential pathway in ischemic stroke may be the occurrence of oxidative stress, i.e., the increased occurrence of reactive oxygen species (ROS) above physiological levels. Oxidative stress has been suggested for many years to cause tissue damage and neuronal death. The toxicity of ROS can be further increased by nitric oxide to produce reactive nitrogen species such as peroxynitrite (ONOO^−^), a molecule that causes oxidation and nitration of tyrosine residues on proteins [8]. Disappointingly, there is no conclusive evidence of a causal link between oxidative stress and the development of disease, and there is no successful therapeutic application targeting oxidative stress. To date, clinical attempts to scavenge ROS by applying antioxidants did not result in clinical benefit [9] or even caused harm [10],[11]. However, the characterization of the relevant enzymatic sources of oxidative stress may allow therapeutic targeting of oxidative stress by preventing the formation of ROS in the first place, instead of scavenging ROS after they have been formed.

A potential source of ROS are NADPH oxidases, the only known enzyme family that is only dedicated to ROS production [12]. Four rodent genes of the catalytic subunit NOX, *Nox1, Nox2, Nox3,* and *Nox4,* have been identified, of which *Nox1*, *Nox2*, and *Nox4* are expressed in the vasculature. NOX4 is the most abundant vascular isoform; its expression is even higher in cerebral than in peripheral blood vessels [13] and, further, induced in stroke [14]. Therefore, we hypothesized that NOX4 is the most relevant source of ROS in stroke.

To test this hypothesis, we generated constitutively NOX4-deficient (*Nox4* ^−/−^) mice and directly compared them to NOX1-deficient (*Nox1^y/^* ^−^) and NOX2-deficient (*Nox2^y/^* ^−^) mice. NOX4 has been implicated in the regulation of systemic and hypoxic vascular responses. Therefore, we had to exclude systemic vascular effects of NOX4 deletion on blood pressure, which may affect stroke outcome independent of a specific neuronal or neurovascular mechanism. Finally, to examine the therapeutic potential of NOX4 as a drug target, we infused the specific NADPH oxidase inhibitor VAS2870 [15] after ischemia, thus mirroring the clinical scenario.

## Results

### NOX4 Is Induced during Ischemic Stroke in Mice and Humans

**Figure 1:**
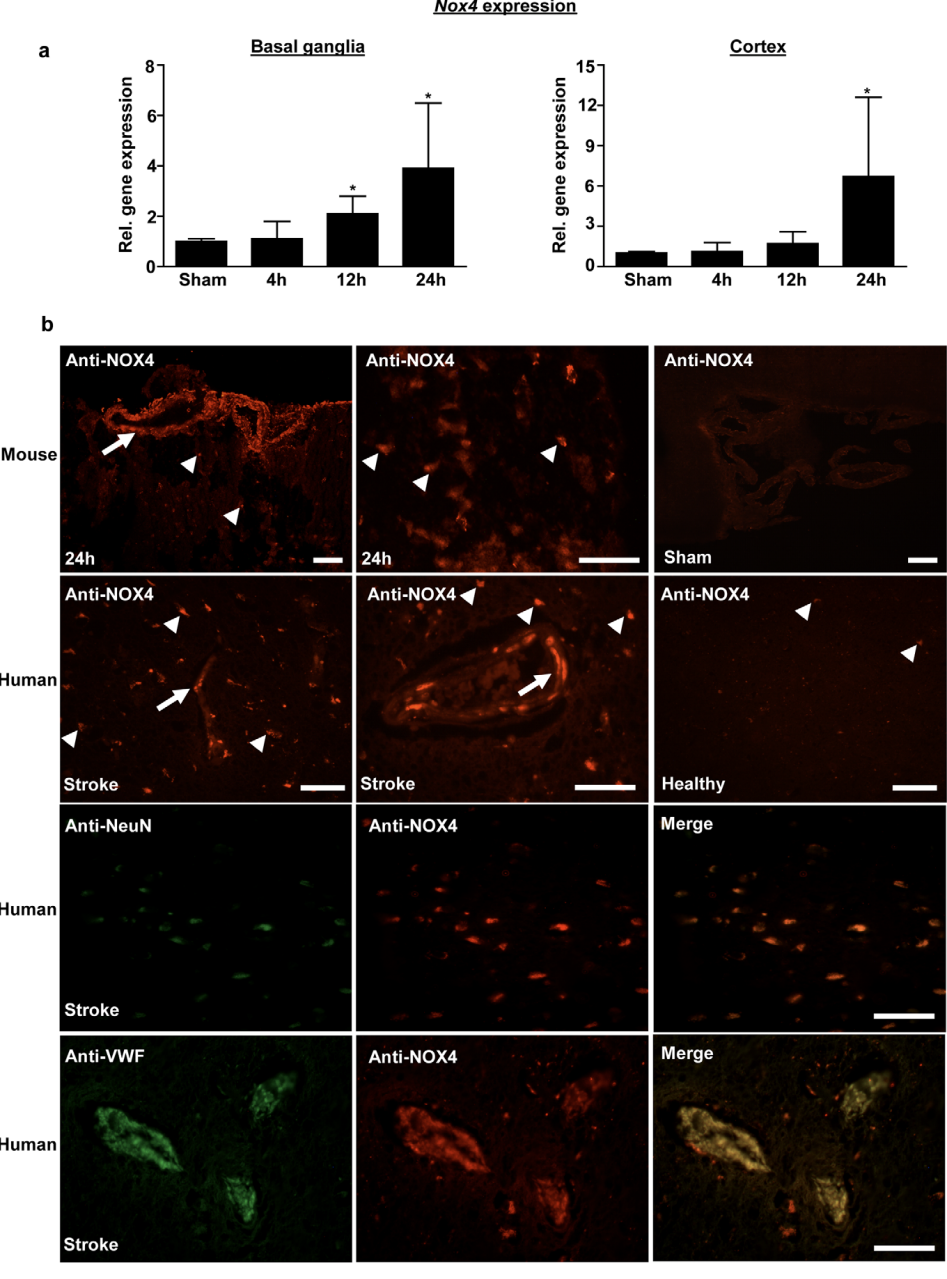
Induction of NOX4 expression after ischemic stroke in mice and humans. (A) Relative gene expression of *Nox4* in the ischemic basal ganglia (left) and cortex (right) of wild-type mice after sham operation and 4 h, 12 h, and 24 h after tMCAO (*n* = 5). *, *p*<0.05, one-way ANOVA, Bonferroni post-hoc test, compared with sham-treated controls. (B) Immunohistochemical detection of NOX4 protein in ischemic brains of wild-type mice (after sham operation or tMCAO, day 1) and humans (samples from stoke patients, after routine autopsy). We compared NOX4 immunolabeling in the ischemic forebrain cortex and the unaffected contralateral side. In ischemic samples, NOX4 was predominantly expressed in neurons (arrowheads) and endothelial cells (arrows). This distribution was confirmed by visualization of NOX4 and NeuN or NOX4 and von Willebrand Factor in the same structures. All scale bars represent 100 µm.

Because NOX4 mRNA is expressed at higher levels in cerebral than in peripheral blood vessels [13] and is induced in stroke [14], we first sought to validate these data not only at the mRNA but also at the protein level. In all experiments, we followed current guidelines defining methodological standards for experimental stroke studies [4],[6],[7],[16],[17]. Here we chose a model of acute ischemic stroke in which mice are subjected to transient middle cerebral artery occlusion (tMCAO). This disease model is thought to involve oxidative stress and an induction of *Nox4* expression [18]. Indeed, expression of NOX4 mRNA was significantly higher 12 h and 24 h after tMCAO in the basal ganglia and neocortex of wild-type mice than in sham-operated controls, in which basal NOX4 expression was low (Figure 1A). This result was validated by immunohistochemistry using a specific NOX4 antibody. We detected a stronger staining in neurons and cerebral blood vessels in wild-type mice subjected to tMCAO than in sham-operated controls. Although immunohistochemistry is not quantitative, this finding suggests higher levels of NOX4 protein (Figure 1B). Importantly, NOX4 staining was also stronger in brain samples from stroke patients. Although NOX4 was barely detectable in healthy brain regions, clear positive labeling of NOX4 was seen in neurons and vascular endothelial cells from the forebrain cortex of stroke patients. This finding was confirmed by double labeling for NeuN (a neuronal marker) or von Willebrand factor (an endothelial marker) and NOX4 in brain tissue (Figure 1B). These data indicate that NOX4 protein is induced during brain ischemia in mice, and this observation would be in agreement with a major functional role for NOX4 in ischemic stroke. Our limited observations in a small number of human cases provide some support to the hypothesis that these processes are also important in human stroke.

### *Nox4* ^−/−^ but Neither *Nox1^y/^* ^−^ nor *Nox2^y/^* ^−^ Mice Are Protected in Both Transient and Permanent Ischemic Stroke

**Figure 2:**
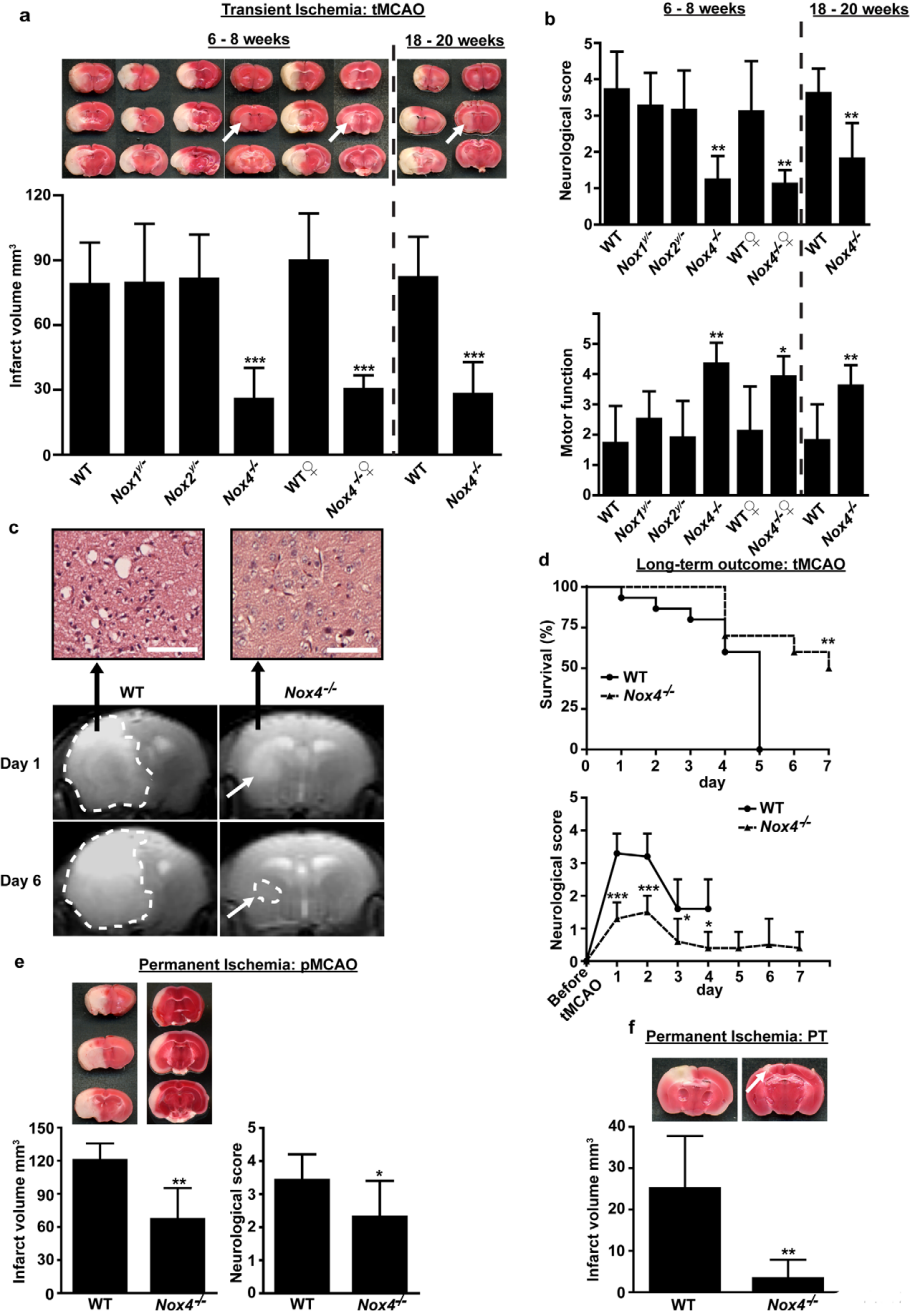
*Nox4* deficiency confers long-term neuroprotection and reduces mortality after acute ischemic stroke in young adult and aged mice of either sex. (A) Upper panel shows representative TTC staining of three corresponding coronal brain sections of 6- to 8-wk-old male and female wild-type (WT) mice, male *Nox1* ^y/−^ mice, male *Nox2* ^y/−^ mice, and male and female *Nox4* ^−/−^ mice, as well as 18- to 20-wk-old male wild-type and *Nox4* ^−/−^ mice on day 1 after tMCAO. The ischemic infarcts (white) appear smallest in the *Nox4* ^−/−^ mice of either age or sex (arrows), and this result was confirmed by infarct volumetry (lower panel). ***, *p*<0.0001, and **, *p*<0.001, one-way ANOVA, Bonferroni post-hoc test compared with wild-type mice (*n* = 8–19 per group). (B) Neurological Bederson score (upper panel) and motor score (lower panel) on day 1 after tMCAO in the eight mouse groups indicated above. (C) Serial magnetic resonance images of cerebral infarcts 1 d and 6 d after tMCAO in wild-type and *Nox4* ^−/−^ mice (lower panel). The broken white lines show hyperintense ischemic lesions on day 1 after tMCAO in wild-type and *Nox4* ^−/−^ mice. Infarcts on day 1 are smaller in *Nox4* ^−/−^ mice than in wild-type mice and remain restricted to the basal ganglia on day 6. Hematoxylin and eosin staining confirmed neuronal damage in the cortex of wild-type mice 24 h after tMCAO (top panel, left), whereas cortical integrity was preserved in *Nox4* ^−/−^ mice (top panel, right). (D) Mortality (upper panel) and long-term functional outcome (Bederson score, lower panel) in 6- to 8-wk-old male *Nox4* ^−/−^ mice and wild-type controls. Survival curve (upper panel): **, *p* = 0.0039, log-rank test compared with wild-type mice (*n* = 10–15 per group). Long-term outcome (lower panel): ***, *p*<0.0001, and *, *p*<0.05, one-way ANOVA, Bonferroni post-hoc test compared with wild-type mice (*n* = 10–15 per group). (E) Upper panel shows representative TTC staining of three corresponding coronal brain sections of 6- to 8-wk-old male wild-type mice (left) and matching *Nox4* ^−/−^ mice (right) on day 1 after pMCAO. Lower panel: Infarct volumes as measured by infarct volumetry (left) and Neurological Bederson score (right). *Nox4* deficiency also protects the brain from permanent ischemia. **, *p*<0.001, and *, *p*<0.05, two-tailed Student's *t*-test compared with wild-type mice (*n* = 7–11 per group). (F) Representative coronal brain sections of wild-type and *Nox4* ^−/−^ mice stained with TTC on day 1 after permanent cortical photothrombosis (PT) (upper panel). Cortical infarctions are smaller in the absence of NOX4 (arrow). The lower panel shows infarct volumes in wild-type and *Nox4* ^−/−^ mice on day 1 after cortical photothrombosis. **, *p*<0.001, two-tailed Student's *t*-test compared with wild-type mice (*n* = 7 per group). All scale bars represent 100 µm.

We first subjected 6- to 8-wk-old male *Nox4* ^−/−^ mice to tMCAO and, after 24 h, assessed infarct volumes by staining brain sections with 2,3,5-triphenyltetrazolium chloride (TTC) (Figure 2A). Infarct volumes were significantly smaller, by approximately 75%, in male *Nox4* ^−/−^ mice than in sex-matched wild-type controls (25.5±14.8 mm^3^ versus 78.7±19.5 mm^3^, respectively). The smaller infarct volume was functionally relevant: compared with wild-type mice, *Nox4* ^−/−^ mice had significantly better overall neurological function (Bederson score 1.2±0.7 in *Nox4* ^−/−^ mice versus 3.7±1.1 in wild-type mice) as well as better basal motor function and coordination (grip test score 4.3±0.7 in *Nox4* ^−/−^ mice versus 1.7±1.3 in wild-type mice) 24 h after tMCAO (Figure 2B). Gender can significantly influence stroke outcome in rodents [4],[16],[17]. Therefore, we also subjected female *Nox4* ^−/−^ mice to 60 min of tMCAO. In line with the results in male mice, *Nox4*-deficient female mice also developed significantly smaller infarctions (30.1±6.7 mm^3^ versus 89.5±22.2 mm^3^, respectively) and less severe neurological deficits (*p*<0.001) than female controls (Figure 2A and 2B). Histological analysis revealed that all infarcts in *Nox4* ^−/−^ mice were restricted to the basal ganglia (arrow in Figure 2A and 2C), whereas in wild-type mice, the neocortex was also consistently affected. Serial magnetic resonance imaging of living mice up to 6 d after stroke showed that in *Nox4* ^−/−^ mice the infarct volume did not increase over time, thus indicating that deletion of the *Nox4* gene provides sustained protection against stroke (Figure 2C). Moreover, infarcts always appeared hyperintense on blood-sensitive constructive interference in steady state (CISS) sequences. Hypointense areas, which typically indicate intracerebral hemorrhage, were absent from *Nox4* ^−/−^ mice and wild-type controls. This finding excludes the possibility of an increased rate of bleeding complications caused by *Nox4* deficiency.

To establish any potential specificity of this function for NOX4 compared to NOX1 and NOX2 in stroke, we carried out identical experiments in 6- to 8-wk-old *Nox1* ^y*/*−^ and *Nox2* ^y*/*−^ mice. However, in contrast to *Nox4* ^−/−^ mice, we observed no protection in these animals, neither in terms of infarct volumes nor on functional outcomes on day 1 after tMCAO, even with large subject sample sizes (*n* = 19 for *Nox2* ^y*/*−^ mice, *p*>0.05; Figure 2A).

Ischemic stroke is usually a disease of the elderly and, consequently, one should verify any stroke-protective effects observed in young adult laboratory animals also in an older cohort [4],[16],[17]. Indeed, 18- to 20-wk-old *Nox4* ^−/−^ mice also developed significantly smaller brain infarctions (27.8±15.1 mm^3^ versus 81.8±19.0 mm^3^, respectively) and less severe neurological deficits than age-matched controls, thereby confirming our results in young animals (Figure 2B). We also determined the functional outcome and mortality of 6- to 8-wk-old male *Nox4* ^−/−^ mice and matched wild-type controls over a longer time period after ischemic stroke (Figure 2D). Five days after 60 min of tMCAO, 15 of 15 wild-type mice (100%) had died, which is in line with previous reports [19]. In contrast, seven of ten *Nox4* ^−/−^ mice (70%) survived until day 5, and five of these were still alive after 1 wk (*p* = 0.0039) (Figure 2D). In line with these findings, *Nox4*-deficient mice showed significantly better Bederson scores than controls over the whole observation period, and neurological deficits remained low until day 7 (Figures 2D and S4).

According to the current experimental stroke guidelines [4],[16],[17], any protective effect also requires evaluation in models of both transient and permanent ischemia. We therefore subjected *Nox4* ^−/−^ mice to filament-induced permanent middle cerebral artery occlusion (pMCAO), a procedure in which no tissue reperfusion occurs (Figure 2E). In the absence of *Nox4,* infarct volumes (66.7±28.6 mm^3^ versus 120.1±15.6 mm^3^, *p*<0.05) and neurological deficits (Bederson score 2.3±1.7 versus 3.4±0.8, *p*<0.05) at day 1 after pMCAO were significantly reduced compared with those in wild-type controls, although to a lesser extent than they were in the tMCAO model (Figures 2E and S5).

Brain infarctions following filament-induced pMCAO are large, and the infarct borders are often not very well defined, which limits the accuracy of any estimation on infarct volumes. We therefore used another model of permanent stroke, cortical photothrombosis, to further verify our findings. Here, the lesions are restricted to the cortex and highly reproducible in size and location. Moreover, photothrombosis has been shown to induce early and profound ROS formation and blood-brain-barrier leakage [20],[21], two key readout parameters of the present investigation. Importantly, photothrombosis-induced infarct volumes were as reduced in *Nox4* ^−/−^ mice relative to wild-type mice (3.3±4.6 mm^3^ versus 25.0±12.8 mm^3^, respectively, a difference of 86.8%; Figure 2F) as they were in the tMCAO model.

### No Apparent Vascular Phenotype of *Nox4* ^−/−^ Mice Other Than in Stroke

Based on the physiological distribution of NOX4 in kidney [22], lung [23], and aorta [24], as well as cell biology data obtained using small interfering RNA approaches [23], one would predict basal phenotypes in a *Nox4* ^−/−^ mouse, such as arterial hypotension, reduced hypoxic pulmonary hypertension, and altered renal function. Importantly, these effects could potentially modulate or interfere with stroke outcome even in the absence of a specific neuronal or neurovascular mechanism. Surprisingly, systemic elimination of *Nox4* did not result in any apparent abnormal vascular phenotype (Text S1; Figures S1 and S2; Table S1). In particular, blood pressure was normal, and hypoxic pulmonary hypertension still occurred despite a 20-fold induction of NOX4 in wild-type animals [23]. In contrast, *Nox1*- and *p47phox-*deficient mice (a *Nox2* subunit) have a lower basal blood pressure, and their blood-pressure response to angiotensin II is reduced [25]–[27]. Our data suggest that any phenotype caused by deleting *Nox4*, unlike those caused by deleting *Nox1* and *Nox2*, would indeed be brain-specific.

### Protection from Ischemic Stroke in *Nox4* ^−/−^ Mice Is a Result of Reduced Oxidative Stress, Neuronal Apoptosis, and Blood-Brain-Barrier Leakage

**Figure 3:**
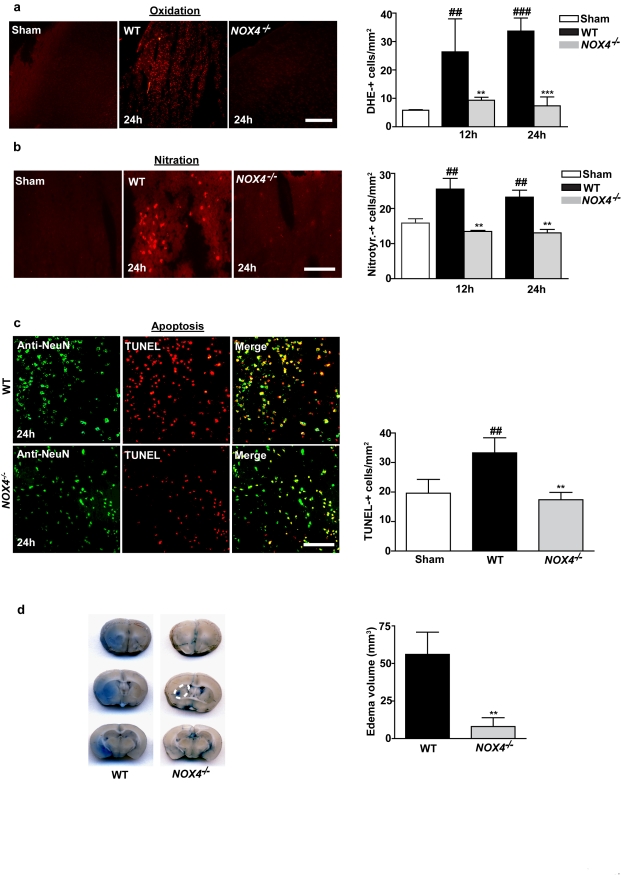
*Nox4* deficiency confers neuroprotection by reducing oxidative stress, neuronal apoptosis, and disruption of the blood–brain barrier. (A and B) Left panels show representative brain sections from sham-operated wild-type (WT) mice and wild-type and *Nox4* ^−/−^ mice 24 h after tMCAO. Sections were stained for ROS and oxidative chemistry using dihydroethidium (DHE) (A), or stained for reactive nitrogen species by using nitrotyrosine (B). Right panels show the number of cells per square millimeter that are positive for ROS or oxidative stress (A) or reactive nitrogen species (B) in the ischemic hemispheres of sham-operated wild-type mice and wild-type and *Nox4* ^−/−^ mice 12 h and 24 h after tMCAO (*n* = 4 per group). (C) Left panels show representative brain sections from sham-operated wild-type mice and wild-type and *Nox4* ^−/−^ mice 24 h after tMCAO, immunolabeled for the neuronal marker NeuN and subjected to TUNEL to show apoptosis. Right panel shows the number of TUNEL-positive neurons per square millimeter in the ischemic hemispheres of sham-operated wild-type mice and wild-type and *Nox4* ^−/−^ mice 24 h after tMCAO (*n* = 4 per group). (D) Left panels show corresponding coronal brain sections of wild-type and *Nox4* ^−/−^ mice on day 1 after tMCAO and injection of Evans blue. Extravasation of Evans blue was reduced in areas where infarcts were regularly present in *Nox4* ^−/−^ mice (basal ganglia, broken white line). The right panel shows the extent of extravasation (i.e., edema volume) as determined by planimetry in the wild-type and *Nox4* ^−/−^ mice 24 h after tMCAO (*n* = 6 per group). For (A–C), ###, *p*<0.0001, and ##, *p*<0.001, compared with sham-treated mice; ***, *p*<0.0001, and **, *p*<0.001, compared with wild-type mice by two-way ANOVA, Bonferroni post-hoc test. For (D), **, *p*<0.001, Two-tailed Student′s *t*-test, compared with wild-type mice. All scale bars represent 100 µm.

Next we sought to elucidate the underlying mechanisms of this NOX4-specific neurotoxicity in stroke. NOX4 can form superoxide or H_2_O_2_, which can interact with nitric oxide to form reactive nitrogen species. Therefore, we stained brain sections with broad-spectrum indicators of oxidative/nitrative stress, i.e., dihydroethidium [28] and nitrotyrosine [8]. At 12 h and 24 h after tMCAO, brains from wild-type mice exhibited a significantly larger amount (by a factor of 2.5–3.5) of ROS in neurons than brains from sham-operated animals, as quantified by dihydroethidium staining (Figure 3A). Neurons from *Nox4* ^−/−^ mice, in contrast, showed only very small ischemia-induced increases in ROS relative to those in sham-operated controls (*p*>0.05). ROS formation from neurons after 24 h was also significantly reduced in *Nox4* ^−/−^ mice subjected to pMCAO (Figure S6). Because the dihydroethidium stain may also indicate oxidative chemistry events, including formation of ONOO^−^ and nitration of protein tyrosine residues [8], we analyzed the extent of protein nitration in *Nox4* ^−/−^ and wild-type mice subjected to tMCAO. In agreement with our findings on the generation of ROS, tissue nitration occurred to a lesser extent in ischemic brains from *Nox4* ^−/−^ mice than in those from wild-type controls (Figure 3B). Oxidative chemistry events such as the formation of ROS and peroxynitrite, as detected by dihydroethidium staining and nitrotyrosine immunolabeling, can induce neuronal apoptosis, which is a well-established mechanism of tissue damage in ischemic stroke [29],[30]. Indeed, superimposed TUNEL and NeuN immunolabeling revealed widespread apoptosis of neurons in wild-type mice 24 h after stroke onset (Figure 3C). In contrast, the number of apoptotic neurons in *Nox4* ^−/−^ mice subjected to tMCAO was significantly lower, and the basal apoptotic turnover rate in *Nox4* ^−/−^ mice fell within the range found in sham-operated mice (*p*>0.05) (Figure 3C).

We also detected NOX4 in cerebral blood vessels (Figure 1B, white arrow indicates endothelial cells). Therefore, we hypothesized that *Nox4* deficiency also influences the disruption of the blood–brain barrier and edema formation mediated by ROS [31]. Integrity of the blood–brain barrier was preserved in *Nox4* ^−/−^ mice on day 1 after tMCAO. This finding correlated with significantly less brain edema in *Nox4* ^−/−^ mice than in wild-type controls, as assessed by the extent of extravasation of Evans blue stain (8.0±5.9 mm^3^ in *Nox4* ^−/−^ mice versus 96.2±5.9 mm^3^ in wild-type mice). Importantly, almost no brain edema was seen in the brain regions where infarcts were regularly present in *Nox4* ^−/−^ mice (basal ganglia; Figure 3D, area delineated by the broken white line). This result indicates that the lesser edema seen in the *Nox4* ^−/−^ mice was a specific phenomenon and mechanistically relevant but was not due to smaller infarct volumes.

### Treatment with the NOX Inhibitor VAS2870 Effectively Protects Ischemic Brain Damage Even When Applied After Stroke

**Figure 4:**
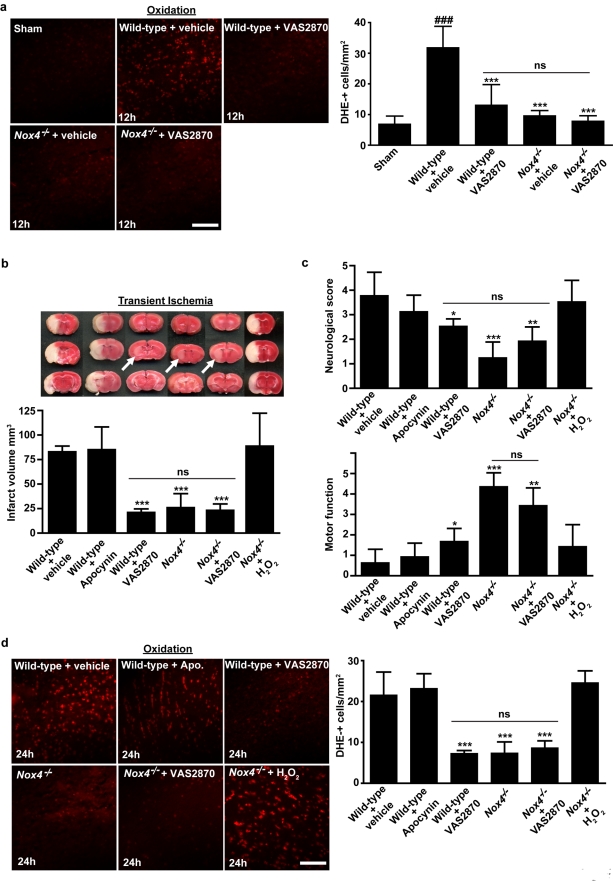
The NADPH oxidase inhibitor VAS2870 protects the brain from damage during acute ischemic stroke. (A) Left panel shows representative images of vital brain slices from sham-operated wild-type (WT) mice and wild-type mice and *Nox4* ^−/−^ mice 12 h after tMCAO. Slices were incubated ex vivo with VAS2870 (10 µM) or carrier solution (1% DMSO; control) for 30 min and stained with dihydroethidium (DHE) to detect ROS. Right panel shows number of ROS-positive cells per square millimeter in brain slices from mice in the different treatment groups (*n* = 5 per group). (B) Upper panel shows representative TTC staining of three corresponding coronal brain sections of wild-type mice treated with (left to right) carrier solution (10% DMSO; control), 100 µg of apocynin intravenously 1 h after tMCAO, or 2 mg of VAS2870 intrathecally 2 h and 12 h after tMCAO, untreated *Nox4* ^−/−^ mice, *Nox4* ^−/−^ mice treated with 2 mg of VAS2870 intrathecally 2 h and 12 h after tMCAO, and *Nox4* ^−/−^ mice treated with H_2_O_2_ intrathecally (15 mg/kg) immediately after the occlusion of the middle cerebral artery and then every hour until 6 h after stroke induction. Ischemic infarcts (white) appear smaller (arrows) in VAS2870-treated wild-type mice and *Nox4* ^−/−^ mice than in control mice, but those in apocynin-treated mice are similar to those in control mice. VAS2870 could not further decrease infarct volumes in *Nox4* ^−/−^ mice. Exogenous H_2_O_2_ reversed the stroke-protective phenotype in *Nox4* ^−/−^ mice. These results were confirmed by infarct volumetry (lower panel) (*n* = 7–10 per group). (C) Neurological Bederson score (upper panel) and motor score (lower panel) on day 1 after tMCAO in the different animal groups indicated in (B) (*n* = 7–10 per group). (D) Left panel shows representative brain sections from the different animal groups indicated in (B) stained for ROS by using dihydroethidium. Right panel shows corresponding number of ROS-positive cells per square millimeter in the ischemic hemispheres (*n* = 3–5 per group). For (A), ###, *p*<0.0001, compared with sham-operated mice, ***, *p*<0.0001 compared with control mice, ns, not significant, one-way ANOVA, Bonferroni post-hoc test. For (B–D), ***, *p*<0.0001, **, *p*<0.001, *, *p*<0.05, ns, not significant, one-way ANOVA, Bonferroni post-hoc test, compared with controls. All scale bars represent 100 µm.

Finally, we wanted to examine whether these genetic insights into the biology of oxidative stress in stroke and the role of NOX4 in general can be translated into a therapeutic intervention. Importantly, this intervention would have to be effective post-stroke and ideally it would be pharmacological. Therefore, we examined the efficacy of a validated, low-molecular-weight NADPH oxidase inhibitor, VAS2870 [15],[32]–[34], in vital brain slices and in vivo. VAS2870 equally inhibits the ROS-generating activity of all NOX subunits, i.e., NOX1, NOX2, and NOX4. Vital brain slices [35] taken from wild-type mice 12 h after tMCAO produced significantly less ROS after pretreatment with 10 µM VAS2870, as did brain slices from untreated *Nox4* ^−/−^ mice (Figure 4A). Importantly, incubating ischemic slices from *Nox4* ^−/−^ mice with VAS2870 had no additional inhibitory effect on superoxide formation (Figure 4A). This finding further underlines the extraordinary role of NOX4 in generating oxidative stress during the course of ischemic stroke, while other NOX isoforms such as NOX1 or NOX2 are obviously less relevant.

To determine whether VAS2870 is also active when applied in vivo, we administered 2 mg of VAS2870 intrathecally to wild-type mice 2 h and 12 h after tMCAO. This experimental therapeutic approach significantly reduced brain infarct volumes (20.7±4.0 mm^3^ in VAS2870-treated mice versus 82.4±6.4 mm^3^ in vehicle-treated controls) and significantly improved neurological function, to the same extent as observed for the deletion of *Nox4* in mice (Figure 4B and 4C). Moreover, less oxidative stress was detected in ischemic brains from VAS2870-treated animals than in those from vehicle-treated controls (Figure 4D). Again, post-stroke application of VAS2870 to *Nox4* ^−/−^ mice had no additive neuroprotective or superoxide-lowering effect compared to the outcomes in wild-type animals treated with VAS2870 or untreated *Nox4* ^−/−^ mice (Figure 4B–4D). This observation is consistent with our ex vivo findings in ischemic brain slices and reaffirms that NOX4 rather than NOX1 or NOX2 is critically involved in the pathophysiology of ischemic stroke. Another, less specific inhibitor that also targets molecules other than NADPH oxidases [36],[37], apocynin, had no effect on infarct size or functional outcome when given post-stroke and did not reduce the formation of ROS in vivo (Figure 4B and 4C).

To further examine whether the neuroprotective effect observed in *Nox4* ^−/−^ mice is specifically related to reduced ROS formation and not due to other nonspecific or developmental defects, we performed a rescue experiment by restoring cerebral ROS levels in *Nox4* ^−/−^ mice during the course of ischemic stroke by applying exogenous H_2_O_2_ (Figure 4B–4D). Indeed, intrathecal administration of H_2_O_2_ rescued the phenotype in *Nox4* ^−/−^ mice, and infarct volumes, functional deficits, and stroke-induced ROS formation returned to the levels observed in wild-type mice (Figure 4B–4D).

## Discussion

Here we identify NOX4 as a relevant molecular source of oxidative stress in cerebral ischemia, including some cases of human stroke. Our data suggest that NOX4-mediated oxidative stress leads to neuronal damage via leakage of the blood–brain barrier and neuronal apoptosis—two pathophysiological hallmarks of ischemic stroke. The extent of neuroprotection conferred by the absence of NOX4 in male and female *Nox4* ^−/−^ mice was exceptional and preserved in old animals. Importantly, the outcomes of these genetic experiments were mimicked when we pharmacologically inhibited NADPH oxidases within a clinically relevant time after induction of stroke. We consider this a key finding for the wider concept of oxidative stress, which might also be of relevance for other disease states, such as neurotrauma and neuroinflammation, where oxidative stress, blood–brain barrier damage, and neurotoxicity are involved. Rather than focusing on antioxidants and the disappointing outcomes of their application, the identification of the relevant source of oxidative stress and preventing its formation may represent an approach with clinical potential.

The hypothesis that free radicals are involved in acute ischemic stroke and account for secondary infarct growth dates back to the 1970s [38] but has remained unproven [38],[39]. The extent of neuroprotection that we observed is exceptional compared with that seen in many other pre-clinical stroke studies, in which the reduction of infarct size usually does not exceed 30%–40% [40]. Such moderate reductions of infarct volume have not translated into improvement of neurological status [3]. Most notably, continuous assessment of functional deficits until 7 d after stroke revealed that *Nox4*-null mice indeed showed a better amplitude rather than simply altered kinetics of recovery. This protection in *Nox4* ^−/−^ mice was further underlined by a significantly reduced post-stroke long-term mortality.

Secondary infarct growth mediated for example by edema formation or hemorrhagic transformation is common during the course of brain ischemia and can lead to worsening of neurological symptoms [39]. Serial magnetic resonance imaging revealed that infarcts in *Nox4* ^−/−^ mice remain small, even at later stages of infarct development, and signs of intracerebral hemorrhage were consistently absent, thus indicating that NOX4 inhibition is likely to be safe and persistently effective.

A plethora of compounds have provided neuroprotection in animal models of brain ischemia, but they all failed in human clinical trials [4]. This translational roadblock has been attributed mainly to inadequate pre-clinical study design and severe methodological shortcomings. Important confounding factors are a lack of randomization or rater-blinded evaluation of study results, and use of only one stroke model [16]. Strictly adhering to current expert recommendations for basic stroke trials, we here demonstrate that in the absence of NOX4, brain tissue can be salvaged after ischemia or reperfusion injury (as occurs in the tMCAO model). Most importantly, neuroprotection was preserved in old male and female *Nox4* ^−/−^ mice as well as in *Nox4* ^−/−^ mice subjected to permanent ischemia (i.e., cortical photothrombosis or pMCAO). Compared to in the tMCAO model, however, the reduction of infarct size in the pMCAO model was less pronounced though still significant. Distinct pathomechanisms that can be positively influenced only in the presence of tissue reperfusion, i.e., after tMCAO but not pMCAO, such as progressive thrombus formation in the cerebral microvasculature [41], might account for this quantitative difference. Indeed, preliminary results suggest that clotting is attenuated in the cerebral vessels of *Nox4* ^−/−^ mice subjected to tMCAO but not pMCAO (unpublished data). Clearly, elimination of NOX4 remains beneficial in the absence of arterial recanalization, a condition frequently observed in human stroke.

In our experiments, deficiency of NOX1 or NOX2 had no impact on infarct size or functional outcome after tMCAO. Although others have described protective effects of NOX2 deficiency after experimental stroke [42]–[44], we could not reproduce those findings. The exact reasons for this discrepancy are unclear at present. Differences in the experimental protocols and middle cerebral artery occlusion times, which varied between 30 min and 120 min in previous investigations, might play a role here [42]–[44]. In contrast to these previous studies, however, we used especially high numbers (*n* = 19) of *Nox2^y/^* ^−^ mice to verify our findings. Moreover, type-II (beta) error of the differences between infarct volumes in *Nox2^y/^* ^−^ mice and wild-type controls was only 7% in our study (93% power, respectively) (Tables S3–S5), which is a very powerful result compared to the positive reports on *Nox2* deficiency in cerebral ischemia [42]–[44] as well as to many other experimental stroke studies in general [4],[45]. Moreover, the fact that VAS2870, which specifically inhibits NADPH oxidases, could not further decrease infarct size and ROS formation in *Nox4* ^−/−^ mice ex vivo and in vivo (Figure 4) clearly argues against a major role of NOX1 or NOX2 in the pathophysiology of acute ischemic stroke. Finally, protein expression levels of NOX1 and NOX2 were almost unchanged in the brains of *Nox4* ^−/−^ mice (Figure S3C), underlining that the profound neuroprotection we observed is mediated by deficiency or blockade of NOX4 itself and not by secondary effects.

Nevertheless, we cannot completely rule out contributions of other sources of ROS. Referring to this, Block et al. recently reported that a functional NOX4 is present and regulated in mitochondria, indicating the existence of a hitherto undescribed source of mitochondrial ROS [46].

An unprecedented need exists for more effective therapies for acute stroke, the second leading cause of death worldwide [1]. We have demonstrated that pharmacological inhibition of NADPH oxidases using the specific NADPH oxidase inhibitor VAS2870 [15],[32]–[34] protects mice from brain ischemia within a clinically meaningful 2-h time window. In contrast, the commonly used organic compound apocynin may not be a NOX inhibitor in vascular cells but rather acts as a nonspecific antioxidant [36]. It also inhibits Rho kinase inhibitor [37], an activity that increases its nonspecific actions. If apocynin inhibits NADPH oxidases at all, it supposedly blocks the migration of the cellular NADPH oxidase complex subunit p47phox to the membrane, thus interfering with assembly of the functional NOX complex [47]. Therefore, it is unlikely to inhibit the NOX4-containing NADPH oxidase, which acts independently of any cytosolic subunits [12]. Indeed, in our experiments, application of apocynin had no effect on the formation of ROS or of functional outcome after experimental stroke in vivo.

In summary, we have demonstrated that NOX4-derived oxidative stress is a crucial player in the pathophysiology of acute ischemic stroke, while *Nox4* deletion does not affect basal vascular or renal function. *Nox4* gene reconstitution experiments in *Nox4* ^−/−^ mice and studies of the effects of different, structurally unrelated NOX inhibitors—should they become available—would be desirable to further substantiate the causality between NOX4 deficiency and protection from cerebral ischemia. Pharmacological inhibition of NADPH oxidases using specific compounds may also pave new avenues for the treatment of ischemic brain injury in humans. Because NADPH oxidase–mediated production of ROS may represent a general mechanism of neurotoxicity, our findings may extend to other ischemic disorders and neurodegenerative or inflammatory diseases. Further studies in relevant disease models are warranted.

## Materials and Methods

Refer to the Text S1 for more detailed methodology. The generation of the *Nox4*-null mice is described in Figure S3.

### Human Specimens

Specimens from patients who had experienced a stroke were collected during routine autopsy at the Department of Neuropathology, University of Würzburg, Germany.

### Stroke Study Design

Detailed study characteristics are provided in Table S2. We strictly followed the recent international expert recommendations for conducting research in mechanism-driven basic stroke studies [4],[6],[7],[16],[17],[40].

### Stroke Models

If not otherwise mentioned, we performed 60 min of tMCAO in 6- to 8-wk-old male mice weighing 20–25 g, as described previously [48],[49]. To exclude age- and gender-specific effects, 18- to 20-wk-old male and 6- to 8-wk-old female mice were used in some subgroups. For pMCAO the occluding filament was left in situ until sacrificing the animals [41].

At 2 h and12 h after the induction of tMCAO, subgroups of wild-type mice or *Nox4* ^−/−^ mice were randomly selected to receive either 2 mg of the NOX-specific inhibitor VAS2870 (Vasopharm GmbH [32],[33]) or carrier solution (10% dimethyl sulfoxide, Sigma) intrathecally, as described previously [50]. In another group, wild-type mice were injected intravenously with 100 µg of apocynin 1 h after the occlusion of the middle cerebral artery. In order to restore ROS levels in *Nox4* ^−/−^ mice, animals received repetitive intrathecal injections of H_2_O_2_ (15 mg/kg) immediately after the occlusion of the middle cerebral artery and then every hour until 6 h after stroke induction.

Cortical photothrombosis was induced in 6- to 8-wk-old wild-type or *Nox4* ^−/−^ mice as described previously [51],[52].

### Stroke Analysis

Stroke analysis was performed as described previously [53],[54]. To determine infarct size, mice were killed 24 h after tMCAO, pMCAO, or cortical photothrombosis. Brains were cut in 2-mm-thick coronal sections using a mouse brain slice matrix (Harvard Apparatus). The slices were stained with 2% TTC (Sigma-Aldrich) to visualize the infarcts. Planimetric measurements (ImageJ software, United States National Institutes of Health), calculating lesion volumes, were corrected for brain edema as described previously [55].

Determination of brain edema using Evans blue dye was performed as described previously [19].

Magnetic resonance imaging was performed repeatedly at 24 h and 6 d after stroke on a 1.5-T magnetic resonance unit (Vision Siemens) as described previously [56]. We used a custom-made dual channel surface coil designed for examining mice (A063HACG; Rapid Biomedical). The imaging protocol comprised a coronal T2-weighted sequence (slice thickness 2 mm) and a blood-sensitive coronal three-dimensional T2-weighted gradient echo CISS (slice thickness 1 mm) sequence. Magnetic resonance images were assessed with respect to infarct morphology and the occurrence of intracerebral bleeding.

### Vital Brain Slices

Vital brain slices from infarcted mouse brains (between –2 mm and –4 mm from bregma) were prepared as described previously [57].

### Quantitative PCR Analysis

After RNA isolation, we quantified NOX4 mRNA expression using real-time PCR and the TaqMan system (TaqMan Gene Expression Arrays for murine NOX4, assay ID Mm00479246_m1, Applied Biosystems), using 18s rRNA (TaqMan Predeveloped Assay Reagents, part number 4319413E, Applied Biosystems) to normalize the amount of sample RNA.

### Histology and Immunohistochemistry

Histology was performed by using formalin-fixed mouse brains on day 1 after tMCAO. Samples were embedded in paraffin and cut into 4-µm-thick sections (0.5 mm anterior from bregma). After deparaffinization and rehydration, tissues were stained with hematoxylin and eosin or Nissl staining solution (Sigma-Aldrich). Immunohistochemical detection of NOX4 was performed on formalin-fixed human brain slices or cryopreserved mouse brain slices. A NOX4-specific primary antibody [58] was applied at a dilution of 1∶200 overnight at 4°C. To identify the cellular origin we performed double staining of NOX4 with the neuronal marker NeuN (1∶1,000) and the endothelial marker von Willebrand Factor (1∶25).

### Oxidative Chemistry Biomarkers

The presence of ROS and other oxidants such as ONOO^−^ was visualized on frozen mouse brain sections 12 h and 24 h after tMCAO or 24 h after pMCAO using dihydroethidium (Sigma; 2 µM stock) staining, as described previously [59], in coronal brain sections taken from identical regions (–0.5 mm from bregma) of sham-operated controls, wild-type and *Nox4* ^−/−^ mice that had undergone stroke, and wild-type mice and *Nox4* ^−/−^ mice treated with VAS2870 or H_2_O_2_.

Immunohistochemical staining for nitrotyrosine to visualize additional reactive nitrogen species was conducted on cryopreserved brain sections taken from identical regions of the mouse brain (–0.5 mm from bregma) 12 h and 24 h after tMCAO, using a polyclonal nitrotyrosine antibody.

Apoptotic neurons in the ischemic hemisphere 24 h after tMCAO were visualized by TUNEL on paraffin-wax-embedded slices, using the TUNEL in situ cell death detection kit, TMR red (Roche). NeuN/TUNEL double staining was performed on cryopreserved brain slices.

### Quantification of Protein Expression

We quantified amounts of NOX1, NOX2, and NOX4 protein in the cortex and basal ganglia by Western blot analysis.

### Statistical Analysis

Data are expressed as mean ± standard deviation and were analyzed statistically using the PrismGraph 4.0 software package (GraphPad Software). In the case of multiple group comparisons, data were tested for Gaussian distribution with the D'Agostino and Pearson omnibus normality test and then analyzed by Bonferroni-corrected one-way ANOVA or two-way ANOVA. Otherwise, the two-tailed Student's *t*-test was applied. For comparison of survival curves the log-rank test was used. *P*-values less than 0.05 were considered significant. Detailed power and type-II (beta) error calculations on infarct volumes are provided in Tables S3–S5.

### Accession Numbers

The GenBank (http://www.ncbi.nlm.nih.gov/Genbank) accession numbers for the genes discussed in this paper are *NOX1,* NM_172203; *NOX2,* NM_007807; and *NOX4,* NM_015760.

## Supporting Information

Figure S1 **Systemic and pulmonary blood pressure as well as kidney function in** ***Nox4*** ^−/−^ **mice are unchanged.** (A and B) Radiotelemetry recordings of basal mean arterial pressure (MAP) and heart rate (HR) of wild-type (WT) (open circles, *n* = 10) and *Nox4* ^−/−^ (filled squares, *n* = 14) mice. Data are represented as 1-h (A) and 24-h (B) averages of mean arterial pressure (left panels) and heart rate (right panels). Dark and light periods are denoted by black and white bars, respectively. (C) Right ventricular systolic pressure (RVSP) as assessed in vivo in anaesthetized *Nox4* ^−/−^ and wild-type mice. (D) Mean pulmonary arterial pressure (PAP) in isolated perfused lungs during normoxic (21% O_2_) ventilation. (E) Strength of hypoxic pulmonary vasoconstriction (HPV) as indicated by the maximum increase in PAP (ΔPAP) upon acute hypoxic ventilation (10 min, 1% O_2_) in isolated perfused lungs. No significant differences were observed between wild-type and *Nox4* ^−/−^ mice. Data are derived from six mice in each case. (F) Renal hypertrophy as assessed by kidney weight per body surface area (BSA) (g/m^2^). There was no significant difference in terms of renal mass between wild-type and *Nox4* ^−/−^ mice at 17 wk of age. (G) Albuminuria at 17 wk of age (µg/24 h). There was no significant difference in 24-h urinary albumin excretion between wild-type and *Nox4* ^−/−^ mice at 17 wk of age.
(1.23 MB TIF)

Figure S2 **Cerebral blood flow, cerebral vasculature, and brain structure are normal in** ***Nox4*** ^−/−^ **mice.** (A) Regional cerebral blood flow (rCBF) in the right territory of the middle cerebral artery as measured by laser Doppler flowmetry in wild-type (WT) mice and in *Nox1* ^y/−^, *Nox2* ^y/−^, and *Nox4* ^−/−^ mice (*n* = 4 per group) at baseline levels, after insertion of the thread (ischemia) and again 10 min after removal of the thread (reperfusion). No significant differences were observed between the groups at any time point. *p*>0.05, two-way ANOVA, Bonferroni post-hoc test, compared with baseline rCBF. (B) Assessment of the cerebral vasculature in wild-type and *Nox4* ^−/−^ mice. A complete circle of Willis (white arrows) was identified in all animals studied, and the distribution of the trunk and branch of the middle cerebral artery appeared to be anatomically identical among the genotypes. (C) Normal brain structure in *Nox4* ^−/−^ mice. Representative Nissl-stained 5-µm coronal paraffin-wax-embedded brain sections of 3-mo-old wild-type and *Nox4* ^−/−^ mice (*n* = 3 each), showing a macroscopic view (uppermost panel), formation of the hippocampus formation (center panel), and somatomotor areas of the neocortex (lowermost panel).
(1.64 MB TIF)

Figure S3 **Generation of** ***Nox4*** **knockout mice and counter-regulation of NOX1 and NOX2.** (A) Construct development for *Nox4* knockout mice. Exons 14 and 15 are flanked by *lox*P sites and followed by a floxed neomycin resistance gene (*neo*) and a negative-selection cassette coding for diphtheria toxin A (*dta*) as described in the Text S1. Embryonic stem cell clones were generated by homologous recombination with the targeting vector. Transient expression of *Cre* recombinase results in three different recombination events. Type 1 results in deletion of the *neo* cassette and thus floxed exons 14 and 15. These cells can be used to generate conditional *Nox4* knockout. Type 2 results in deletion of the floxed exons, and type 3 results in the deletion of exons 14 and 15 and the *neo* cassette. These cells were used to generate the *Nox4* knockout mice. (B) Western blot demonstrating the absence of the 64-kDa NOX4 band in the aorta, lung, and kidney of *Nox4* ^−/−^ mice. (C) Expression of NOX1 and NOX2 is not upregulated in *Nox4* ^−/−^ mice. The uppermost left panel shows results of densitometric analysis of the NOX1 134-kDa band in brain samples of the cortex and basal ganglia from *Nox4* ^−/−^ (pale bar) and wild-type mice (black bar). Data are presented as the relative amount of the NOX1 band normalized to GAPDH and represent the mean ± standard error of three samples. The right panel shows a Western blot comparison of brain and aorta samples from wild-type mice demonstrating the presence of the 134-kDa band in both samples. The center and lowest panels show results of densitometric analysis of the 91- and 53-kDa NOX2 bands seen in brain samples from the cortex and basal ganglia of *Nox4* ^−/−^ (pale bar) and wild-type mice (black bar). Data are presented as the relative amount of either the 91-kDa band or 53 k-Da band normalized to GAPDH and represent the mean ± standard error of three samples. The bottom right panel shows a Western blot comparison of NOX2 expression in the brain and aorta of wild-type mice, demonstrating the presence of the 91-kDa and 53-kDa bands in both tissues.
(24.75 MB TIF)

Figure S4 **Long-term outcomes are improved in** ***Nox4*** ^−/−^ **mice after tMCAO.** Long-term outcome of motor function (grip test) in 6- to 8-wk-old male *Nox4* ^−/−^ mice (*n* = 10) and wild-type (WT) controls (*n* = 15) after tMCAO. *Nox4* ^−/−^ mice performed better over the whole observation period. **, *p*<0.001 and *, *p*<0.05, one-way ANOVA, Bonferroni post-hoc test compared with wild-type mice.
(0.27 MB TIF)

Figure S5 **Motor function after pMCAO.** Motor function was assessed by the grip test in 6- to 8-wk-old male *Nox4* ^−/−^ mice (*n* = 7) and wild-type (WT) controls (*n* = 11) 24 h after pMCAO. Two-tailed Student's *t*-test compared with wild-type mice. ns, not significant.
(0.18 MB TIF)

Figure S6 **Oxidative stress is reduced in brains from** *Nox4* ^−/−^ **mice after pMACO.** Left panels show representative brain sections from wild-type (WT) and *Nox4* ^−/−^ mice 24 h after sham operation of pMCAO. Sections were stained for ROS and oxidative chemistry using dihydroethidium. Right panel shows the number of cells per square millimeter that are positive for ROS or oxidative stress in the ischemic hemisphere of wild-type and *Nox4* ^−/−^ mice 24 h after sham operation or pMCAO (*n* = 3–5 per group). ##, *p*<0.001 compared with sham-treated mice; **, *p*<0.001 compared with wild-type mice by one-way ANOVA, Bonferroni post-hoc test.
(1.76 MB TIF)

Table S1 **Results of blood gas analysis and posterior communicating artery (PComA) score in wild-type and *Nox4*** ^−/−^ **mice.**
(0.04 MB PDF)

Table S2 **Stroke study population.**
(0.09 MB PDF)

Table S3 **Power and type-II (beta) error calculations on infarct volumes depicted in** **Figure 2A** **.**
(0.06 MB PDF)

Table S4 **Power and type-II (beta) error calculations on infarct volumes depicted in** **Figure 2E** **.**
(0.05 MB PDF)

Table S5 **Power and type-II (beta) error calculations on infarct volumes depicted in** **Figure 4B** **.**
(0.06 MB PDF)

Text S1 **Supplementary results, supplementary methods, and supplementary references.**
(0.32 MB DOC)

## Abbreviations

CISS: constructive interference in steady state
KO: knock out
pMCAO: permanent middle cerebral artery occlusion
ROS: reactive oxygen species
rt-PA: recombinant tissue plasminogen activator
tMCAO: transient middle cerebral artery occlusion
TTC: 2,3,5-triphenyltetrazolium chloride
WT: wild type

## References

[1] World Health Organization (2008). The top ten causes of death. Fact sheet number 310.. http://www.who.int/mediacentre/factsheets/fs310_2008.pdf.

[2] Elkins J. S, Johnston S. C (2003). Thirty-year projections for deaths from ischemic stroke in the United States.. Stroke.

[3] O'Collins V. E, Macleod M. R, Donnan G. A, Horky L. L, van der Worp B. H (2006). 1,026 experimental treatments in acute stroke.. Ann Neurol.

[4] Dirnagl U (2006). Bench to bedside: the quest for quality in experimental stroke research.. J Cereb Blood Flow Metab.

[5] Whalley K (2006). Slicing into stroke therapeutics.. Nat Rev Drug Discov.

[6] Sena E. S, van der Worp H. B, Bath P. M, Howells D. W, Macleod M. R (2010). Publication bias in reports of animal stroke studies leads to major overstatement of efficacy.. PLoS Biol.

[7] van der Worp H. B, Howells D. W, Sena E. S, Porritt M. J, Rewell S (2010). Can animal models of disease reliably inform human studies?. PLoS Med.

[8] Eliasson M. J, Huang Z, Ferrante R. J, Sasamata M, Molliver M. E (1999). Neuronal nitric oxide synthase activation and peroxynitrite formation in ischemic stroke linked to neural damage.. J Neurosci.

[9] Steinhubl S. R (2008). Why have antioxidants failed in clinical trials?. Am J Cardiol.

[10] Dotan Y, Pinchuk I, Lichtenberg D, Leshno M (2009). Decision analysis supports the paradigm that indiscriminate supplementation of vitamin E does more harm than good.. Arterioscler Thromb Vasc Biol.

[11] Omenn G. S (2007). Chemoprevention of lung cancers: lessons from CARET, the beta-carotene and retinol efficacy trial, and prospects for the future.. Eur J Cancer Prev.

[12] Opitz N, Drummond G. R, Selemidis S, Meurer S, Schmidt H. H (2007). The ‘A’s and ‘O’s of NADPH oxidase regulation: a commentary on “Subcellular localization and function of alternatively spliced Noxo1 isoforms”.. Free Radic Biol Med.

[13] Miller A. A, Drummond G. R, Schmidt H. H, Sobey C. G (2005). NADPH oxidase activity and function are profoundly greater in cerebral versus systemic arteries.. Circ Res.

[14] McCann S. K, Dusting G. J, Roulston C. L (2008). Early increase of Nox4 NADPH oxidase and superoxide generation following endothelin-1-induced stroke in conscious rats.. J Neurosci Res.

[15] Niethammer P, Grabher C, Look A. T, Mitchison T. J (2009). A tissue-scale gradient of hydrogen peroxide mediates rapid wound detection in zebrafish.. Nature.

[16] Fisher M, Feuerstein G, Howells D. W, Hurn P. D, Kent T. A (2009). Update of the stroke therapy academic industry roundtable preclinical recommendations.. Stroke.

[17] Macleod M. R, Fisher M, O'Collins V, Sena E. S, Dirnagl U (2009). Good laboratory practice: preventing introduction of bias at the bench.. Stroke.

[18] Vallet P, Charnay Y, Steger K, Ogier-Denis E, Kovari E (2005). Neuronal expression of the NADPH oxidase NOX4, and its regulation in mouse experimental brain ischemia.. Neuroscience.

[19] Austinat M, Braeuninger S, Pesquero J. B, Brede M, Bader M (2009). Blockade of bradykinin receptor B1 but not bradykinin receptor B2 provides protection from cerebral infarction and brain edema.. Stroke.

[20] Watson B. D, Dietrich W. D, Busto R, Wachtel M. S, Ginsberg M. D (1985). Induction of reproducible brain infarction by photochemically initiated thrombosis.. Ann Neurol.

[21] Kleinschnitz C, Bendszus M, Frank M, Solymosi L, Toyka K. V (2003). In vivo monitoring of macrophage infiltration in experimental ischemic brain lesions by magnetic resonance imaging.. J Cereb Blood Flow Metab.

[22] Geiszt M, Kopp J. B, Varnai P, Leto T. L (2000). Identification of renox, an NAD(P)H oxidase in kidney.. Proc Natl Acad Sci U S A.

[23] Mittal M, Roth M, Konig P, Hofmann S, Dony E (2007). Hypoxia-dependent regulation of nonphagocytic NADPH oxidase subunit NOX4 in the pulmonary vasculature.. Circ Res.

[24] Griendling K. K (2004). Novel NAD(P)H oxidases in the cardiovascular system.. Heart.

[25] Gavazzi G, Banfi B, Deffert C, Fiette L, Schappi M (2006). Decreased blood pressure in NOX1-deficient mice.. FEBS Lett.

[26] Landmesser U, Cai H, Dikalov S, McCann L, Hwang J (2002). Role of p47(phox) in vascular oxidative stress and hypertension caused by angiotensin II.. Hypertension.

[27] Matsuno K, Yamada H, Iwata K, Jin D, Katsuyama M (2005). Nox1 is involved in angiotensin II-mediated hypertension: a study in Nox1-deficient mice.. Circulation.

[28] Veresh Z, Racz A, Lotz G, Koller A (2008). ADMA impairs nitric oxide-mediated arteriolar function due to increased superoxide production by angiotensin II-NAD(P)H oxidase pathway.. Hypertension.

[29] Bobba A, Atlante A, Moro L, Calissano P, Marra E (2007). Nitric oxide has dual opposite roles during early and late phases of apoptosis in cerebellar granule neurons.. Apoptosis.

[30] Loh K. P, Huang S. H, De Silva R, Tan B. K, Zhu Y. Z (2006). Oxidative stress: apoptosis in neuronal injury.. Curr Alzheimer Res.

[31] Sandoval K. E, Witt K. A (2008). Blood-brain barrier tight junction permeability and ischemic stroke.. Neurobiol Dis.

[32] Stielow C, Catar R. A, Muller G, Wingler K, Scheurer P (2006). Novel Nox inhibitor of oxLDL-induced reactive oxygen species formation in human endothelial cells.. Biochem Biophys Res Commun.

[33] ten Freyhaus H, Huntgeburth M, Wingler K, Schnitker J, Baumer A. T (2006). Novel Nox inhibitor VAS2870 attenuates PDGF-dependent smooth muscle cell chemotaxis, but not proliferation.. Cardiovasc Res.

[34] Lange S, Heger J, Euler G, Wartenberg M, Piper H. M (2009). Platelet-derived growth factor BB stimulates vasculogenesis of embryonic stem cell-derived endothelial cells by calcium-mediated generation of reactive oxygen species.. Cardiovasc Res.

[35] Meuth S. G, Budde T, Kanyshkova T, Broicher T, Munsch T (2003). Contribution of TWIK-related acid-sensitive K+ channel 1 (TASK1) and TASK3 channels to the control of activity modes in thalamocortical neurons.. J Neurosci.

[36] Heumuller S, Wind S, Barbosa-Sicard E, Schmidt H. H, Busse R (2008). Apocynin is not an inhibitor of vascular NADPH oxidases but an antioxidant.. Hypertension.

[37] Schluter T, Steinbach A. C, Steffen A, Rettig R, Grisk O (2008). Apocynin-induced vasodilation involves Rho kinase inhibition but not NADPH oxidase inhibition.. Cardiovasc Res.

[38] Flamm E. S, Demopoulos H. B, Seligman M. L, Poser R. G, Ransohoff J (1978). Free radicals in cerebral ischemia.. Stroke.

[39] Dirnagl U, Iadecola C, Moskowitz M. A (1999). Pathobiology of ischaemic stroke: an integrated view.. Trends Neurosci.

[40] Crossley N. A, Sena E, Goehler J, Horn J, van der Worp B (2008). Empirical evidence of bias in the design of experimental stroke studies: a metaepidemiologic approach.. Stroke.

[41] Pham M, Kleinschnitz C, Helluy X, Bartsch A. J, Austinat M (2010). Enhanced cortical reperfusion protects coagulation factor XII-deficient mice from ischemic stroke as revealed by high-field MRI.. Neuroimage.

[42] Chen H, Song Y. S, Chan P. H (2009). Inhibition of NADPH oxidase is neuroprotective after ischemia-reperfusion.. J Cereb Blood Flow Metab.

[43] Jackman K. A, Miller A. A, De Silva T. M, Crack P. J, Drummond G. R (2009). Reduction of cerebral infarct volume by apocynin requires pretreatment and is absent in Nox2-deficient mice.. Br J Pharmacol.

[44] Walder C. E, Green S. P, Darbonne W. C, Mathias J, Rae J (1997). Ischemic stroke injury is reduced in mice lacking a functional NADPH oxidase.. Stroke.

[45] van der Worp H. B, de Haan P, Morrema E, Kalkman C. J (2005). Methodological quality of animal studies on neuroprotection in focal cerebral ischaemia.. J Neurol.

[46] Block K, Gorin Y, Abboud H. E (2009). Subcellular localization of Nox4 and regulation in diabetes.. Proc Natl Acad Sci U S A.

[47] Touyz R. M (2008). Apocynin, NADPH oxidase, and vascular cells: a complex matter.. Hypertension.

[48] Elvers M, Stegner D, Hagedorn I, Kleinschnitz C, Braun A (2010). Impaired alpha(IIb)beta(3) integrin activation and shear-dependent thrombus formation in mice lacking phospholipase D1.. Sci Signal.

[49] Berna-Erro A, Braun A, Kraft R, Kleinschnitz C, Schuhmann M. K (2009). STIM2 regulates capacitive Ca2+ entry in neurons and plays a key role in hypoxic neuronal cell death.. Sci Signal.

[50] Wu W. P, Xu X. J, Hao J. X (2004). Chronic lumbar catheterization of the spinal subarachnoid space in mice.. J Neurosci Methods.

[51] Kleinschnitz C, Braeuninger S, Pham M, Austinat M, Nolte I (2008). Blocking of platelets or intrinsic coagulation pathway-driven thrombosis does not prevent cerebral infarctions induced by photothrombosis.. Stroke.

[52] Schroeter M, Jander S, Stoll G (2002). Non-invasive induction of focal cerebral ischemia in mice by photothrombosis of cortical microvessels: characterization of inflammatory responses.. J Neurosci Methods.

[53] Kleinschnitz C, Hofstetter H. H, Meuth S. G, Braeuninger S, Sommer C (2006). T cell infiltration after chronic constriction injury of mouse sciatic nerve is associated with interleukin-17 expression.. Exp Neurol.

[54] Kleinschnitz C, Pozgajova M, Pham M, Bendszus M, Nieswandt B (2007). Targeting platelets in acute experimental stroke: impact of glycoprotein Ib, VI, and IIb/IIIa blockade on infarct size, functional outcome, and intracranial bleeding.. Circulation.

[55] Ginsberg M. D, Becker D. A, Busto R, Belayev A, Zhang Y (2003). Stilbazulenyl nitrone, a novel antioxidant, is highly neuroprotective in focal ischemia.. Ann Neurol.

[56] Kleinschnitz C, De Meyer S. F, Schwarz T, Austinat M, Vanhoorelbeke K (2009). Deficiency of von Willebrand factor protects mice from ischemic stroke.. Blood.

[57] Meuth S. G, Kleinschnitz C, Broicher T, Austinat M, Braeuninger S (2009). The neuroprotective impact of the leak potassium channel TASK1 on stroke development in mice.. Neurobiol Dis.

[58] Anilkumar N, Weber R, Zhang M, Brewer A, Shah A. M (2008). Nox4 and nox2 NADPH oxidases mediate distinct cellular redox signaling responses to agonist stimulation.. Arterioscler Thromb Vasc Biol.

[59] Murakami K, Kondo T, Kawase M, Li Y, Sato S (1998). Mitochondrial susceptibility to oxidative stress exacerbates cerebral infarction that follows permanent focal cerebral ischemia in mutant mice with manganese superoxide dismutase deficiency.. J Neurosci.

