# Supplementary figures and images for: Post-Stroke Inhibition of Induced NADPH Oxidase Type 4 Prevents Oxidative Stress and Neurodegeneration
Source: PLoS Biol. 2010 Sep 21;8(9):e1000479. doi: 10.1371/journal.pbio.1000479 (PMC2943442; doi:10.1371/journal.pbio.1000479)

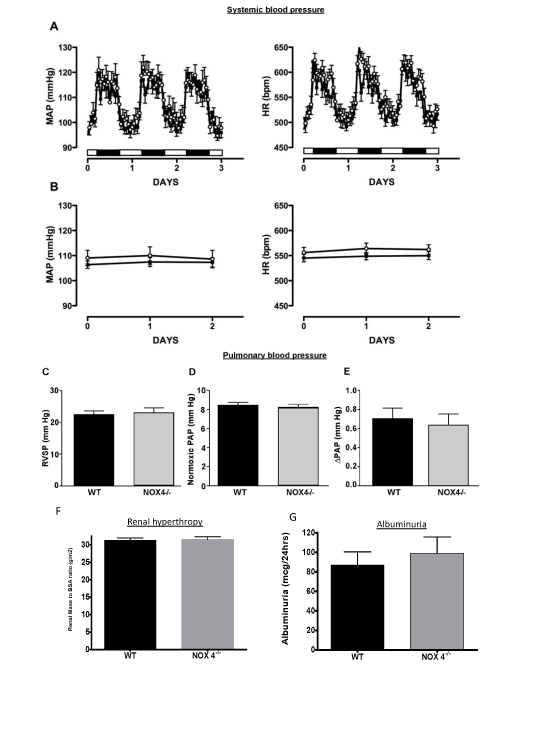

Supplement: Figure S1 — Systemic and pulmonary blood pressure as well as kidney function in Nox4 −/− mice are unchanged. (A and B) Radiotelemetry recordings of basal mean arterial pressure (MAP) and heart rate (HR) of wild-type (WT) (open circles, n = 10) and Nox4 −/− (filled squares, n = 14) mice. Data are represented as 1-h (A) and 24-h (B) averages of mean arterial pressure (left panels) and heart rate (right panels). Dark and light periods are denoted by black and white bars, respectively. (C) Right ventricular systolic pressure (RVSP) as assessed in vivo in anaesthetized Nox4 −/− and wild-type mice. (D) Mean pulmonary arterial pressure (PAP) in isolated perfused lungs during normoxic (21% O2) ventilation. (E) Strength of hypoxic pulmonary vasoconstriction (HPV) as indicated by the maximum increase in PAP (ΔPAP) upon acute hypoxic ventilation (10 min, 1% O2) in isolated perfused lungs. No significant differences were observed between wild-type and Nox4 −/− mice. Data are derived from six mice in each case. (F) Renal hypertrophy as assessed by kidney weight per body surface area (BSA) (g/m2). There was no significant difference in terms of renal mass between wild-type and Nox4 −/− mice at 17 wk of age. (G) Albuminuria at 17 wk of age (µg/24 h). There was no significant difference in 24-h urinary albumin excretion between wild-type and Nox4 −/− mice at 17 wk of age. (1.23 MB TIF) [file pbio.1000479.s001.tif]

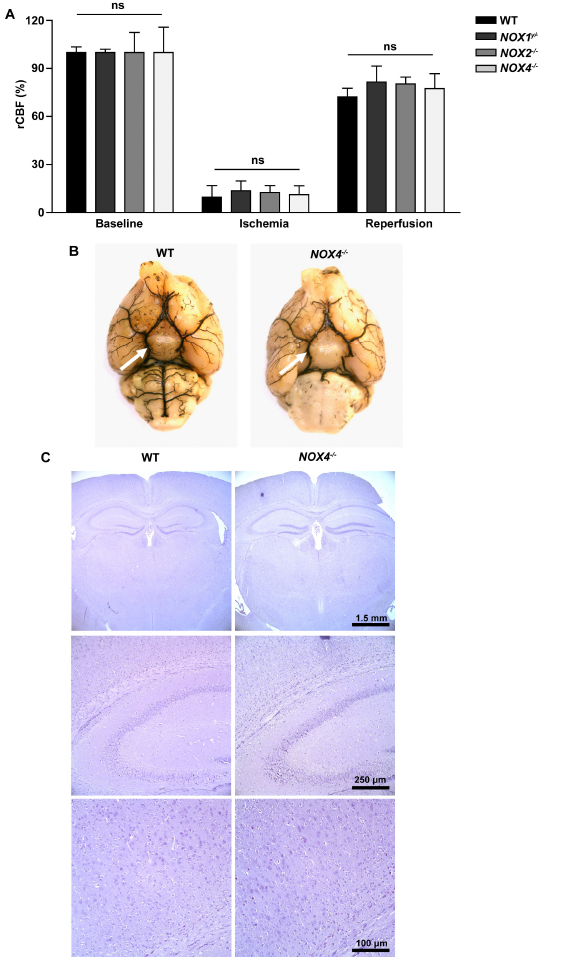

Supplement: Figure S2 — Cerebral blood flow, cerebral vasculature, and brain structure are normal in Nox4 −/− mice. (A) Regional cerebral blood flow (rCBF) in the right territory of the middle cerebral artery as measured by laser Doppler flowmetry in wild-type (WT) mice and in Nox1 y/−, Nox2 y/−, and Nox4 −/− mice (n = 4 per group) at baseline levels, after insertion of the thread (ischemia) and again 10 min after removal of the thread (reperfusion). No significant differences were observed between the groups at any time point. p>0.05, two-way ANOVA, Bonferroni post-hoc test, compared with baseline rCBF. (B) Assessment of the cerebral vasculature in wild-type and Nox4 −/− mice. A complete circle of Willis (white arrows) was identified in all animals studied, and the distribution of the trunk and branch of the middle cerebral artery appeared to be anatomically identical among the genotypes. (C) Normal brain structure in Nox4 −/− mice. Representative Nissl-stained 5-µm coronal paraffin-wax-embedded brain sections of 3-mo-old wild-type and Nox4 −/− mice (n = 3 each), showing a macroscopic view (uppermost panel), formation of the hippocampus formation (center panel), and somatomotor areas of the neocortex (lowermost panel). (1.64 MB TIF) [file pbio.1000479.s002.tif]

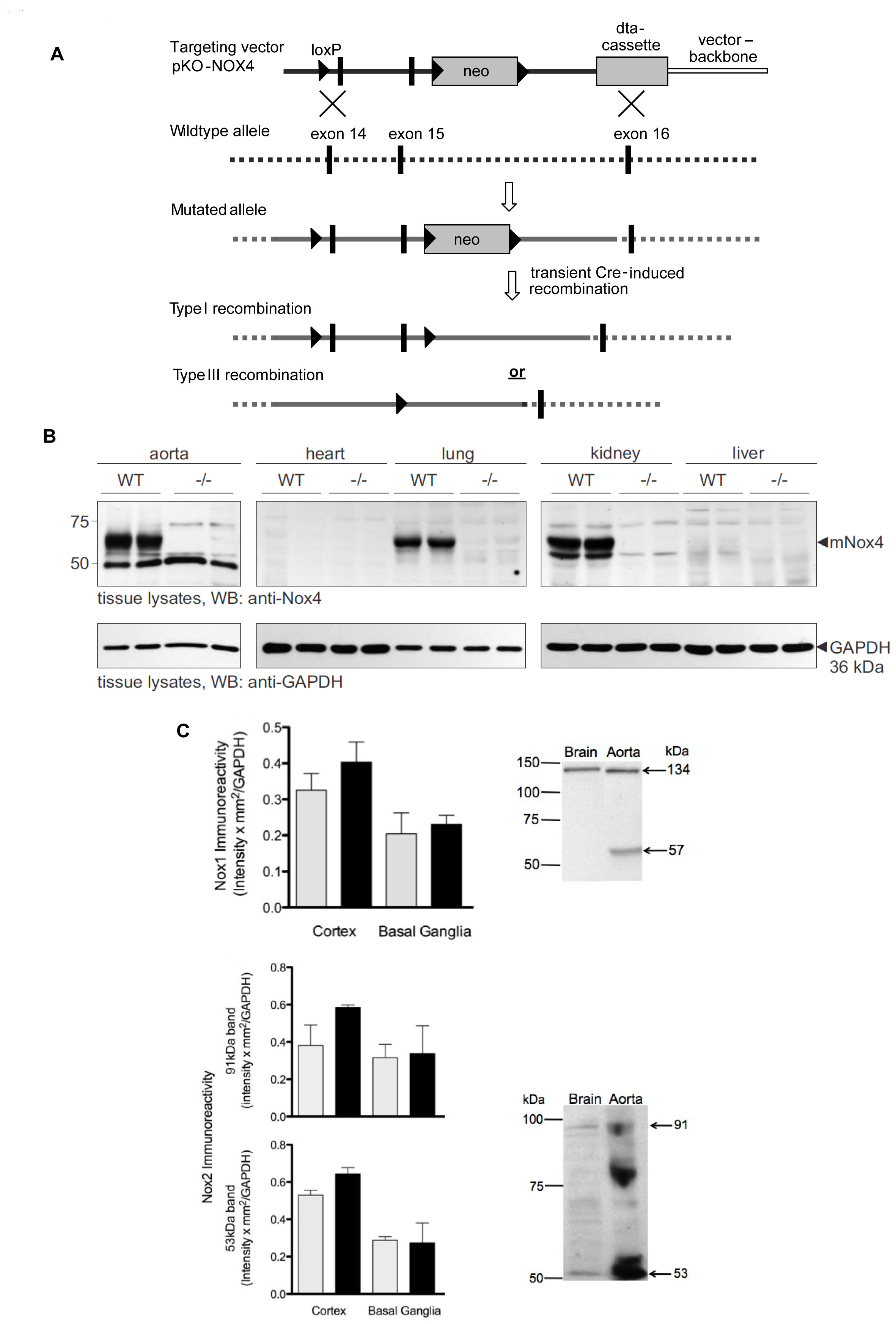

Supplement: Figure S3 — Generation of Nox4 knockout mice and counter-regulation of NOX1 and NOX2. (A) Construct development for Nox4 knockout mice. Exons 14 and 15 are flanked by loxP sites and followed by a floxed neomycin resistance gene (neo) and a negative-selection cassette coding for diphtheria toxin A (dta) as described in the Text S1. Embryonic stem cell clones were generated by homologous recombination with the targeting vector. Transient expression of Cre recombinase results in three different recombination events. Type 1 results in deletion of the neo cassette and thus floxed exons 14 and 15. These cells can be used to generate conditional Nox4 knockout. Type 2 results in deletion of the floxed exons, and type 3 results in the deletion of exons 14 and 15 and the neo cassette. These cells were used to generate the Nox4 knockout mice. (B) Western blot demonstrating the absence of the 64-kDa NOX4 band in the aorta, lung, and kidney of Nox4 −/− mice. (C) Expression of NOX1 and NOX2 is not upregulated in Nox4 −/− mice. The uppermost left panel shows results of densitometric analysis of the NOX1 134-kDa band in brain samples of the cortex and basal ganglia from Nox4 −/− (pale bar) and wild-type mice (black bar). Data are presented as the relative amount of the NOX1 band normalized to GAPDH and represent the mean ± standard error of three samples. The right panel shows a Western blot comparison of brain and aorta samples from wild-type mice demonstrating the presence of the 134-kDa band in both samples. The center and lowest panels show results of densitometric analysis of the 91- and 53-kDa NOX2 bands seen in brain samples from the cortex and basal ganglia of Nox4 −/− (pale bar) and wild-type mice (black bar). Data are presented as the relative amount of either the 91-kDa band or 53 k-Da band normalized to GAPDH and represent the mean ± standard error of three samples. The bottom right panel shows a Western blot comparison of NOX2 expression in the brain and aorta of wild-type mice, d [file pbio.1000479.s003.tif]

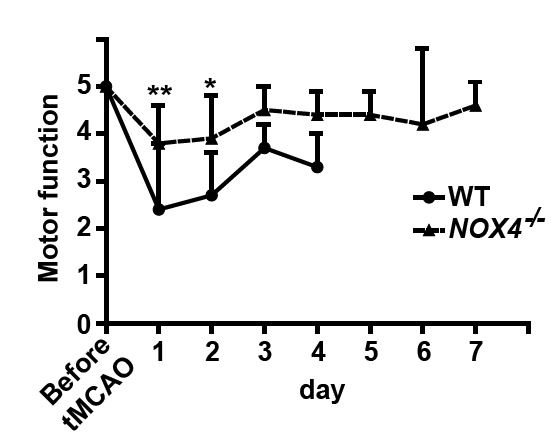

Supplement: Figure S4 — Long-term outcomes are improved in Nox4 −/− mice after tMCAO. Long-term outcome of motor function (grip test) in 6- to 8-wk-old male Nox4 −/− mice (n = 10) and wild-type (WT) controls (n = 15) after tMCAO. Nox4 −/− mice performed better over the whole observation period. **, p<0.001 and *, p<0.05, one-way ANOVA, Bonferroni post-hoc test compared with wild-type mice. (0.27 MB TIF) [file pbio.1000479.s004.tif]

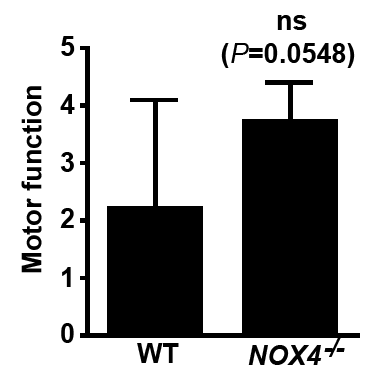

Supplement: Figure S5 — Motor function after pMCAO. Motor function was assessed by the grip test in 6- to 8-wk-old male Nox4 −/− mice (n = 7) and wild-type (WT) controls (n = 11) 24 h after pMCAO. Two-tailed Student's t-test compared with wild-type mice. ns, not significant. (0.18 MB TIF) [file pbio.1000479.s005.tif]

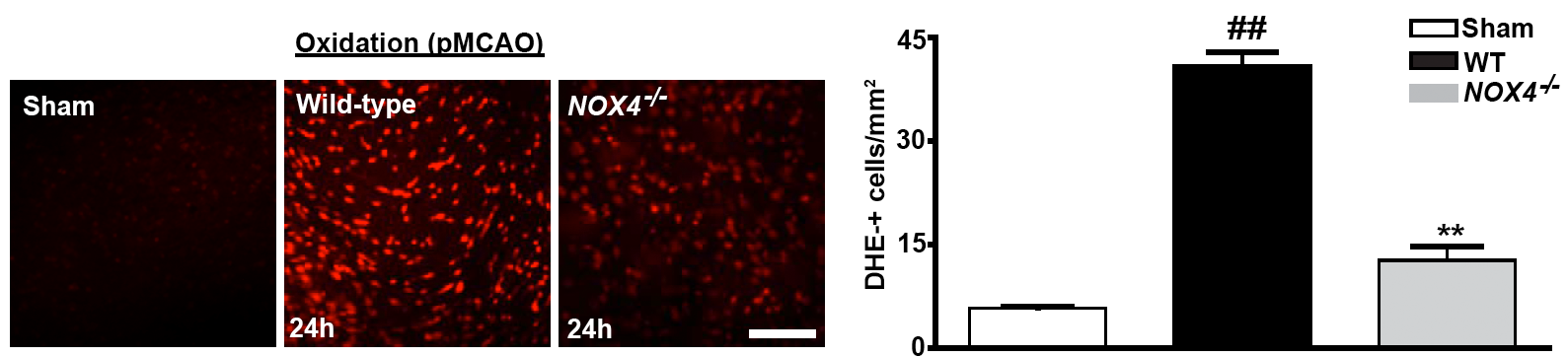

Supplement: Figure S6 — Oxidative stress is reduced in brains from Nox4 −/− mice after pMACO. Left panels show representative brain sections from wild-type (WT) and Nox4 −/− mice 24 h after sham operation of pMCAO. Sections were stained for ROS and oxidative chemistry using dihydroethidium. Right panel shows the number of cells per square millimeter that are positive for ROS or oxidative stress in the ischemic hemisphere of wild-type and Nox4 −/− mice 24 h after sham operation or pMCAO (n = 3–5 per group). ##, p<0.001 compared with sham-treated mice; **, p<0.001 compared with wild-type mice by one-way ANOVA, Bonferroni post-hoc test. (1.76 MB TIF) [file pbio.1000479.s006.tif]
