# Supplementary material for: Post-Stroke Inhibition of Induced NADPH Oxidase Type 4 Prevents Oxidative Stress and Neurodegeneration
Source: PLoS Biol. 2010 Sep 21;8(9):e1000479. doi: 10.1371/journal.pbio.1000479 (PMC2943442; doi:10.1371/journal.pbio.1000479)
Supplement: Table S1 — Results of blood gas analysis and posterior communicating artery (PComA) score in wild-type and Nox4 −/− mice. (0.04 MB PDF) [file pbio.1000479.s007.pdf]

Table S1 Results of blood gas analysis and posterior communicating artery (PComA) score in wild-type and *NOX4*$^{-/-}$ mice.

	Wild-type (*n* = 4)	*NOX4*$^{-/-}$ (*n* = 4)	*P*
**PaO_2 (mmHg)**	73.3 ± 8.0	80.2 ± 16.9	ns
**$PaCO_2$ (mmHg)**	49.1 ± 6.7	51.3 ± 3.79	ns
**pH**	7.21 ± 0.08	7.21 ± 0.04	ns
**PComA score**	1.3 ± 1.0	1.7 ± 1.1	ns
