# Supplementary material for: Post-Stroke Inhibition of Induced NADPH Oxidase Type 4 Prevents Oxidative Stress and Neurodegeneration
Source: PLoS Biol. 2010 Sep 21;8(9):e1000479. doi: 10.1371/journal.pbio.1000479 (PMC2943442; doi:10.1371/journal.pbio.1000479)
Supplement: Table S2 — Stroke study population. (0.09 MB PDF) [file pbio.1000479.s008.pdf]

Table S2 Stroke study popluation

Strain/ genotype	Procedure	Age/sex	Randomized, n	Drop-outs, n (reason)	Mortality rate, n (%)	Analyzed, n (%)	Read-out (number of mice)
C57BL/6 mice	tMCAO	6–8 weeks/♂	96	5 (SAH)<br>3 (BS = 0)<br>2 (apnea during surgery)<br>Subtotal 10/96 (10.4%)	6/96 (6.3)	86/96 (89.6)	Infarct volume (11), including<br>▸ Functional scores day 1 (11)<br>▸ Regional cerebral blood flow (4)<br>▸ Blood gases (4)<br>Long-term functional scores/survival (15)<br>Magnetic resonance imaging (5)<br>Brain edema (6)<br>Histology/histochemistry (24)<br>Brain slice preparations (10)<br>Real-time RT-PCR (15)
		18–20 wk/♂	10	0/10 (0%)	0/10 (0%)	10/10 (100%)	Infarct volume (10), including<br>▸ Functional scores (10)
		6–8 wk/♀	10	1 (SAH)<br>Subtotal 1/10 (10%)	1/10 (10.0%)	9/10 (90%)	Infarct volume (9), including<br>▸ Functional scores (9)
	PT	6–8 weeks/♂	8	1 (apnea during surgery)<br>Subtotal 1/8 (12.5%)	1/8 (12.5%)	7/8 (87.5%)	Infarct volume (7)
	pMCAO	6–8 weeks/♂	12	1 (SAH)<br>Subtotal 1/12 (8.3%)	1/12 (8.3%)	11/12 (91.6%)	Infarct volume (11), including<br>▸ Functional scores day 1 (11)<br>▸ Histology/histochemistry (5)
	None	6–8 weeks/♂	9	0/9 (0%)	0/9 (0%)	9/9 (100%)	Cerebral vasculature (3)<br>Nissl stain (3)<br>Western blot (3)
C57BL/6 mice	tMCAO + VAS2870 i.t.	6–8 weeks/♂	10	0/10 (0%)	0/10 (0%)	10/10 (100%)	Infarct volume (7), including<br>▸ Functional scores (7)<br>Histology/histochemistry (3)
C57BlL6 mice	tMCAO + DMSO i.t.	6–8 weeks/♂	10	0/10 (0%)	0/10 (0%)	10/10 (100%)	Infarct volume (7), including<br>▸ Functional scores (7)<br>Histology/histochemistry (3)
C57BL/6 mice	tMCAO + apocynin i.v.	6–8 weeks/♂	17	1 (SAH)<br>1 (apnea during surgery)<br>Subtotal 2/17 (11.8%)	2/17 (11.8%)	15/17 (88.2%)	Infarct volume (10), including<br>▸ Functional scores (10)<br>Histology/histochemistry (5)

C57BL/6 mice	Sham operation	6–8 weeks/♂	26	0/26 (0%)	0/26 (0%)	26/26 (100%)	Histology/histochemistry (16)<br>Brain slice preparations (5)<br>Real-time RT-PCR (5)
NOX1$^{y/–}$ mice	tMCAO	6–8 weeks/♂	11	1 (SAH)<br>1 (BS = 0)<br>Subtotal 2/11 (18.2%)	1/11 (9.1%)	9/11 (81.8)	Infarct volume (9), including<br>▸ Functional scores (9)<br>▸ Regional cerebral blood flow (4)
NOX2$^{–/–}$ mice	tMCAO	6–8 weeks/♂	21	2 (SAH)<br>Subtotal 2/21(9.5%)	2/21 (4.8%)	19/21 (90.5%)	Infarct volume (19), including<br>▸ Functional scores (9)<br>▸ Regional cerebral blood flow (4)
NOX4$^{–/–}$ mice	tMCAO	6–8 weeks/♂	59	2 (SAH)<br>2 (BS = 0)<br>1 (apnea during surgery)<br>Subtotal 5/59 (8.5%)	3/59 (5.1%)	54/59 (91.5%)	Infarct volume (10), including<br>▸ Functional scores (10)<br>▸ Regional cerebral blood flow (4)<br>▸ Blood gases (4)<br>Magnetic resonance imaging (5)<br>Brain edema (6)<br>Histology/histochemistry (20)<br>Brain slice preperations (13)
		18–20 wk/♂	10	0/10 (0%)	0/10 (0%)	10/10 (100%)	Infarct volume (10), including<br>▸ Functional scores (10)
		6–8 wk/♀	7	0/7 (0%)	0/7 (0%)	7/7 (100%)	Infarct volume (7)
	PT	6–8 weeks/♂	7	0/7 (0%)	0/7 (0%)	7/7 (100%)	Infarct volume (7)
	pMCAO	6–8 weeks/♂	9	1 (SAH)<br>1 (apnea during surgery)<br>Subtotal 2/9 (22.2%)	1/9 (11.1%)	7/9 (77.8%)	Infarct volume (7), including<br>▸ Functional scores day 1 (7)<br>▸ Histology/histochemistry (5)
	None	6–8 weeks/♂	9	0/9 (0%)	0/9 (0%)	9/9 (100%)	Cerebral vasculature (3)<br>Nissl stain (3)<br>Western blot (3)
NOX4$^{–/–}$ mice	tMCAO + VAS2870 i.t.	6–8 weeks/♂	8	1 (apnea during surgery)<br>Subtotal 1/8 (12.5%)	1/8 (12.5%)	7/8 (87.5%)	Infarct volume (7), including<br>▸ Functional scores (7)<br>▸ Histology/histochemistry (3)
NOX4$^{–/–}$ mice	tMCAO + H_2O_2 i.t.	6–8 weeks/♂	10	1 (apnea during surgery)<br>Subtotal 1/10 (10%)	1/10 (10%)	9/10 (90%)	Infarct volume (9), including<br>▸ Functional scores (9)<br>▸ Histology/histochemistry (5)
**Total**			**359**	**28/359 (7.8%)**	**20/359 (5.6%)**	**331/359 (92.5%)**	

Abbreviations: BS, Bederson score; DMSO, dimethyl sulfoxide (10%); i.v., intravenously; i.t., intrathecally; PT, photothrombosis; RT-PCR, reverse transcription polymerase chain reaction; SAH, subarachnoid hemorrhage; t/pMCAO, transient/permanent middle cerebral artery occlusion
