# Supplementary material for: Post-Stroke Inhibition of Induced NADPH Oxidase Type 4 Prevents Oxidative Stress and Neurodegeneration
Source: PLoS Biol. 2010 Sep 21;8(9):e1000479. doi: 10.1371/journal.pbio.1000479 (PMC2943442; doi:10.1371/journal.pbio.1000479)
Supplement: Table S3 — Power and type-II (beta) error calculations on infarct volumes depicted in Figure 2A . (0.06 MB PDF) [file pbio.1000479.s009.pdf]

Table S3 Power and type-II (beta) error calculations on infarct volumes as depicted in Figure 2a.

	***NOX1*$^{-/-}$ 6-8 wk♂**	***NOX2*$^{-/-}$ 6-8 wk♂**	***NOX4*$^{-/-}$ 6-8 wk♂**	***NOX4*$^{-/-}$ 18-20 wk♂**	***NOX4*$^{-/-}$ 6-8 wk♀**
N	9	19	10	10	7
SD (mm^3)	27.6	20.8	14.5	26.7	6.7
Delta (mm^3)	27.7	27.7	27.7	28.7	31.3
**Power (%)**	**70**	**93**	**93**	**93**	**91**
**Type II error (%)**	**30**	**7**	**7**	**7**	**9**

Abbreviations: N, animal numbers; SD, standard deviation; wk, weeks.
