# Supplementary material for: Post-Stroke Inhibition of Induced NADPH Oxidase Type 4 Prevents Oxidative Stress and Neurodegeneration
Source: PLoS Biol. 2010 Sep 21;8(9):e1000479. doi: 10.1371/journal.pbio.1000479 (PMC2943442; doi:10.1371/journal.pbio.1000479)
Supplement: Table S4 — Power and type-II (beta) error calculations on infarct volumes depicted in Figure 2E . (0.05 MB PDF) [file pbio.1000479.s010.pdf]

Table S4 Power and type-II (beta) error calculations on infarct volumes as depicted in Figure 2e.

	NOX4 $^{-/-}$
N	10
SD (mm^3)	14.5
Delta (mm^3)	42.0
**Power (%)**	**97**
**Type II error (%)**	**3**

Abbreviations: N, animal numbers; SD, standard deviation.
