# Supplementary material for: Post-Stroke Inhibition of Induced NADPH Oxidase Type 4 Prevents Oxidative Stress and Neurodegeneration
Source: PLoS Biol. 2010 Sep 21;8(9):e1000479. doi: 10.1371/journal.pbio.1000479 (PMC2943442; doi:10.1371/journal.pbio.1000479)
Supplement: Table S5 — Power and type-II (beta) error calculations on infarct volumes depicted in Figure 4B . (0.06 MB PDF) [file pbio.1000479.s011.pdf]

Table S5 Power and type-II (beta) error calculations on infarct volumes as depicted in Figure 4b.

	**WT Apocynin**	**WT VAS2870**	***NOX4*$^{-/-}$**	***NOX4*$^{-/-}$ VAS2870**	***NOX4*$^{-/-}$ H_2O_2**
N	10	7	10	7	9
SD (mm^3)	23.7	4.0	14.5	6.9	33.9
Delta (mm^3)	28.7	28.7	28.7	28.7	28.7
**Power (%)**	**82**	**99**	**99**	**99**	**70**
**Type II error (%)**	**18**	**1**	**1**	**1**	**30**

Abbreviations: N, animal numbers; SD, standard deviation; WT, wild-type.
