# Supplementary material for: Post-Stroke Inhibition of Induced NADPH Oxidase Type 4 Prevents Oxidative Stress and Neurodegeneration
Source: PLoS Biol. 2010 Sep 21;8(9):e1000479. doi: 10.1371/journal.pbio.1000479 (PMC2943442; doi:10.1371/journal.pbio.1000479)
Supplement: Text S1 — Supplementary results, supplementary methods, and supplementary references. (0.32 MB DOC) [file pbio.1000479.s012.doc]

## Text S1

**Post-stroke inhibition of induced NADPH oxidase type 4 prevents oxidative stress and neurodegeneration**

Christoph Kleinschnitz^1^*, Henrike Grund^2^, Kirstin Wingler^2,3,10^, Melanie E. Armitage^3^, Emma Jones^3^, Manish Mittal^2^, David Barit^4^, Tobias Schwarz^1^, Peter Kraft^1^, Christian Geis^1^, Konstanze Barthel^5^, Michael K. Schuhmann^1^, Alexander M. Herrmann^1^, Sven G. Meuth^1^, Guido Stoll^1^, Sabine Meurer^3^, Anja Schrewe^7^, Lore Becker^7,8^, Valérie Gailus-Durner^7^, Helmut Fuchs^7^, Thomas Klopstock^8^, Martin Hrabě de Angelis^7,9^,Karin Jandeleit-Dahm^4^, Ajay M. Shah^6^, Norbert Weissmann^2^ & Harald H.H.W. Schmidt^2,3,10^*

**1** Neurologische Klinik und Poliklinik, Universität Würzburg, Würzburg, Germany,

**2** Rudolf-Buchheim-Institut für Pharmakologie & Medizinische Klinik, Justus-Liebig-Universität, Gießen, Germany,

**3** Department of Pharmacology and Centre for Vascular Health, Monash University, Melbourne, Australia,

**4** Department of Pharmacology and Toxicology and Cardiovascular Research Institute Maastricht (CARIM), Maastricht University, The Netherlands,

**5** National Stroke Research Institute, Florey Neuroscience Institutes, Melbourne, Australia,

**6** Baker IDI Heart and Diabetes Institute, Juvenile Diabetes Research Foundation (JDRF) International Center for Diabetic Complications Research, Melbourne, Australia,

**7** Abteilung Neurologie, Georg-August Universität Göttingen, Göttingen, Germany,

**8** Universitätsklinik Münster, Klinik und Poliklinik für Neurologie—Entzündliche Erkrankungen des Nervensystems und Neuroonkologie, Münster, Germany,

**9** Institute of Experimental Genetics, Helmholtz Zentrum München, German Research Center for Environmental Health, München, Germany,

**10** Friedrich-Baur-Institut an der Neurologischen Klinik, Klinikum der Ludwig-Maximilians-Universität München, München, Germany, 11 Lehrstuhl für Experimentelle Genetik, Technische Universita¨t München, Freising-Weihenstephan, Germany,

**12** King’s College, London School of Medicine, The James Black Centre, Cardiovascular Division, London, United Kingdom

*To whom correspondence should be addressed. E-mail: h.schmidt@farmaco.unimaas.nl or [christoph.kleinschnitz@mail.uni-wuerzburg.de](mailto:christoph.kleinschnitz@mail.uni-wuerzburg.de)

### Supplementary Results

### Systemic blood pressure, renal and pulmonary functions are unchanged in *NOX4*^–/–^ mice

Despite the prominent expression of NOX4 in blood vessels, systemic blood pressure was surprisingly unchanged in *NOX4*^–/–^ compared with wild-type mice. Both mean arterial pressure (MAP) and heart rate (HR; **Figure S1A**) showed typical circadian variation in all mice, with highest and lowest values corresponding to dark and light phases, respectively. There was no significant difference in 24 h averages of MAP or HR (**Figure S1B**) between wild-type and *NOX4*^–/–^ mice, thus suggesting that *NOX4* deletion has no significant influence on basal MAP or HR in these animals.

NOX4 is also highly expressed in the kidney and lung. However, pulmonary blood pressure under basal and hypoxic conditions as well as basal renal function were surprisingly unchanged in *NOX4*^–/–^ mice compared with wild-type mice. Determination of pulmonary hemodynamics revealed no significant difference when quantifying right ventricular systolic pressure (RVSP) *in vivo* and pulmonary arterial pressure (PAP) in isolated, blood-free, perfused and ventilated lungs (**Figure S1C** and **Figure S1D**). In this setup, PAP is a direct measure of pulmonary vascular resistance. Moreover, the strength of the pulmonary vasoconstrictor response induced by acute hypoxic ventilation was similar in wild-type and *NOX4*^–/–^ mice (**Figure S1E**). We did not observe any significant difference in renal mass between wild-type and *NOX4*^–/–^ mice at the age of 17 weeks (**Figure S1F**). In addition, 24 h urinary excretion of albumin in 17 week-old *NOX4*^–/–^ mice was similar to that in age-matched wild-type mice (**Figure S1G**).

### Basal anatomy and other physiological parameters of *NOX4*^–/–^ mice

To exclude the possibility that differences in muscle strength between wild-type and *NOX4*^–/–^ mice influence motor performance upon stroke, grip strength was measured by using a force-meter (Bioseb, France) with a custom-build grid that allows measurement of the maximum force applied by the mouse when it is slowly pulled away [1,2]. Three measurements each were performed consecutively with the fore paws and then with all 4 paws, in healthy wild-type and *NOX4*^–/–^ mice (n = 10 per group). No significant genotype-related differences in mean grip strength were detected (not shown).

To assess the cerebral vasculature, we examined the circle of Willis and main arteries. A complete circle of Willis was identified in all animals studied. The distribution of the trunk and branch of the middle cerebral artery appeared to be anatomically identical among the genotypes (**Figure S2B**). To further quantitatively examine the vascular structures, we examined the development of the posterior communicating arteries (PComAs), which can affect brain sensitivity to ischemia [3]. The mean score of PComAs in both hemispheres showed no significant differences between wild-type mice and *NOX4*^–/–^ mice (**Table S1**).

We used laser Doppler flowmetry to monitor cerebral blood flow in the tMCAO model [4] in either genotype before surgery (baseline), immediately after tMCAO, and 10 min after removal of the occluding monofilament (reperfusion). Cerebral perfusion did not differ between any group or time point (**Figure S2A**). Furthermore, arterial blood gases showed no significant differences (**Table S1**).

Power and type-II (beta) error calculations (Figure 2A)

For power and type-II (beta) error calculations on infarct volumes after tMCAO (Figure 2A) GraphPad Stat Mate 2.0 software package was used (GraphPad Software, Inc, La Jolla, CA, USA). The mean infarct volume at day 1 after 60 min tMCAO in 6-8 weeks old male wild-type mice was ~ 79.0 mm^3^ or ~ 82.0 mm^3^ in 18-20 weeks old male wild-type mice, respectively (Figure 2A). Infarctions in 6-8 weeks old female wild-type mice at day 1 after 60 min tMCAO were ~ 89.5 mm^3^ in size. We assumed that a ≥ 35% reduction (Delta_1_ = 27.7 mm^3^, Delta_2_ = 28.7 mm^3^, Delta_3_ = 31.3 mm^3^) of infarct size would be of biological relevance [5,6]. The significance level (alpha) was chosen as 0.05 (two-tailed). Given that premises the power to detect a difference between means of infarct volumes of 27.7 mm^3^, 28.7 mm^3^ or 31.3 mm3 ranged between 70% and 93% in our study which is a favourable result compared to many other experimental stroke studies [6,7]. Or in other words: type-II (beta) error ranged between 7% and 30%. Detailed results are given in Table S3.

Power and type-II (beta) error calculations (Figure 2E)

For power and type-II (beta) error calculations on infarct volumes after pMCAO (Figure 2E) GraphPad Stat Mate 2.0 software package was used (GraphPad Software, Inc, La Jolla, CA, USA). The mean infarct volume at day 1 after pMCAO in 6-8 weeks old male wild-type mice was ~ 120.0 mm^3^ (Figure 2E). We assumed that a ≥ 35% reduction (Delta = 42.0 mm^3^) of infarct size would be of biological relevance [5,6]. The significance level (alpha) was chosen as 0.05 (two-tailed). Given that premises the power to detect a difference between means of infarct volumes of 42.0 mm^3^ was 97% in our study, which is a favourable result compared to many other experimental stroke studies [6,7]. Or in other words: type-II (beta) error was 3%. Detailed results are given in Table S4.

**Power and type-II (beta) error calculations (Figure 4B)**

For power and type-II (beta) error calculations on infarct volumes after tMCAO (Figure 4b) GraphPad Stat Mate 2.0 software package was used (GraphPad Software, Inc, La Jolla, CA, USA). The mean infarct volume at day 1 after 60 min tMCAO in vehicle-treated wild-type mice was ~ 82.0 mm^3^ (Figure 4B). We assumed that a ≥ 35% reduction (Delta = 28.7 mm^3^) of infarct size would be of biological relevance [5,6]. The significance level (alpha) was chosen as 0.05 (two-tailed). Given that premises the power to detect a difference between means of infarct volumes of 28.7 mm^3^ ranged between 70% and 99% in our study, which is a favourable result compared to many other experimental stroke studies [7,8]. Or in other words: type-II (beta) error ranged between 1% and 30%. Detailed results are given in Table S5.

## Supplementary Methods

## Materials

The NADPH oxidase inhibitor, VAS2870, was obtained from Vasopharm GmbH (Würzburg, Germany). It was diluted as previously described [9,10,11]. Anti-NOX1 and -NOX4 antibodies were generated and applied as described previously [12,13,14,15,16]. Other antibodies were obtained commercially: anti-NOX2 antibody was from BD Transduction Laboratories (Franklin Lakes, NJ, USA; # 611415); polyclonal nitrotyrosine antibody was from Upstate (Schwalbach, Germany, # 06-284); von Willebrand factor (vWF) antibody was from Dako (Germany; monoclonal mouse anti-human vWF, clone F8/86 code NR. M616, lot 094); mouse neuN monoclonal antibody was from Millipore (MAB377 clone A60; Millipore, Billerica, USA); Alexa594-conjugated goat anti-rabbit secondary antibody was from Molecular Probes (Eugene, Oregon, USA; cat: A-11007) and Alexa488-labeled goat anti-mouse antibody was from Invitrogen (Karlsruhe, Germany); secondary antibody linked to Cy3 (donkey anti-rabbit Cy3) was from Dianova (Hamburg, Germany; # 711-165-152). The TUNEL *in situ* death detection kit, TMR red, was from Roche (Switzerland). H_2_O_2_ was from Merck (Darmstadt, Germany).

### Generation of *NOX4*^–/–^ mice

To directly assess NOX4 function *in vivo*, we disrupted the *NOX4* gene in mice by deleting the NADPH binding pockets located in exons 14 and 15 (**Figure S3**). These *NOX4* knockout mice were generated by Murinus GmbH (Hamburg, Germany). The mouse was designed by using a *Cre*/*lox* recombination technique, which allows creation of either constitutive (type III recombination) or conditional (type I recombination) knockout alleles. The targeting vector was based on the Litmus28 vector (New England Biolabs). It carried exons 14 and 15 flanked by *lox*P sites. For embryonic stem (ES) cell clone selection, a neomycin resistance (*neo*) cassette with a flanking *lox*P site and a diphtheria toxin A (*dta*) cassette for negative selection were included (**Figure S3A**). The *NOX4* exons and the *neo* cassette were located inside homologous regions corresponding to the *NOX4* gene. The *dta* cassette was located outside the homologous region. The targeting construct was brought into E14 embryonic stem cells (derived from the inbred mouse strain 129/Ola with agouti fur color) by electroporation and recombined in the genome by using the homologous regions. This resulted in the replacement of the genomic copies of exons 14 and 15. Recombinant stem cell clones were selected for neomycin resistance and *dta* sensitivity. The correct localization was verified by PCR, and the number of chromosomes was checked. After transient *Cre*-recombinase induced recombination, parts of the *lox*P-flanked regions were removed from the genome. Different products could be identified (**Figure S3A**). Type I recombination removed the neomycin cassette, leaving an intact *NOX4* gene with two additional *lox*P sites in intronic regions. Type III recombination removed exons 14 and 15 as well as the *neo* cassette. Type I deletion is used for generation of conditional knockout mice; type III is a constitutive knockout. All experiments referring to *NOX4*^–/–^ animals were carried out with type III recombination products, from which exons 14 and 15 have been deleted. The ES cell clones were cultivated on fibroblast cells and injected into blastocysts derived from C57Bl/6 mice (with black fur color). The blastocysts were re-injected into pseudo-pregnant foster mothers, which had been mated with vasectomized males. Chimera developing from those blastocysts were examined for skin color. Animals showing large agouti areas were mated with C57Bl/6 females. The F1 offspring was tested for heterozygosity by using PCR. F1 males were mated with two C57Bl/6 females each. Of the resulting offspring, 10 males were genotyped by microsatellite analysis of 110 different polymorphic markers (Charles River MAX-BAX^SM^ analysis). Males showing the highest homology to the wild-type C57Bl/6 strain were mated again. After the content of the recipient (C57Bl/6) genome reached > 95%, the mice were intercrossed to gain homozygous offspring.

All animals were kept under SPF conditions. For routine genotyping, a PCR strategy using tail-snip DNA was used with a combination of three primers, designed to amplify a 435 bp, wild-type PCR fragment and a 517 bp knockout PCR signal (TGT CTG TCG GCG CAC TCA CTA; AGA CAT CCA ATC ATT CCA GTG G; GTG GAT CAA GAA ACA TGC TGA C). For all experiments littermate wild-type mice were used as controls.

### Ethics

A total of 359 mice were used in this study. All procedures for animal handling and experiments were performed in accordance with protocols approved by the Animal Ethics Committees of Monash University (Australian Code of Practice for the Care and Use of Animals for Scientific Purposes), of the University of Gießen (Regierungspräsidium Gießen, Gießen, Germany), of the University of Würzburg (Institutional Review Board of the University of Würzburg, Germany), and by the Government of Upper Bavaria (Regierung von Oberbayern).

## Human stroke specimens

Human stroke specimens were from 4 patients (2 men, 2 women; median age 61.5 years) who had suffered acute ischemic stroke. Infarcted tissue from the frontal and parietal lobe, thalamus, and putamen was used in this study, and sections from the unaffected contralateral regions served as controls. The time interval between stroke onset and death ranged between 24 h and 36 h, and the time between death and removal of the brain was between 12 h and 24 h.

## Stroke study design

Wild-type mice and mice deficient in *NOX1*, *NOX2*, or *NOX4* were randomly assigned to the two operators (C.K. and T.S.) by an independent person not involved in data acquisition and analysis (S.G.M.) in a 1:1 ratio. We used the same randomization protocol for sham-treated mice or mice receiving VAS2870 or H_2_O_2_. We performed surgery and evaluation of all read-out parameters while blinded to the experimental groups. Important anatomical and physiological parameters relevant to stroke development were determined before or throughout surgery. Key findings, e.g. reduction of infarct size in *NOX4*^–/–^ mice or mice treated with VAS2870, were replicated once.

The following conditions excluded mice from end-point analyses (exclusion criteria):

1. Death within 24 h of tMCAO
2. Subarachnoidal hemorrhage (SAH) (as assessed during brain sampling or by MRI)
3. Bederson score = 0 (24 h after tMCAO)

## Stroke models

Operation time per animal did not exceed 15 min. Briefly, mice were anesthetized with 2.5% enflurane (Abbott, Wiesbaden, Germany) in a 70% N_2_O, 30% O_2_ mixture. Core body temperature was maintained at 37°C throughout surgery by using a feedback-controlled heating device. Following a midline skin incision in the neck, the proximal common carotid artery and the external carotid artery were ligated, and a standardized silicon rubber-coated 6.0 nylon monofilament (6021; Doccol Corp., CA, USA) was inserted and advanced via the right internal carotid artery to occlude the origin of the right middle cerebral artery. The intraluminal suture was left *in situ* for 60 min. Then animals were re-anesthetized, and the occluding monofilament was withdrawn to allow reperfusion. For pMCAO the occluding filament was left *in situ* until [sacrificing](http://www.dict.cc/englisch-deutsch/sacrificing.html) the animals [17]. After recovery from anesthesia and after 24 hours, neurological deficits were scored by two blinded investigators and quantified according to Bederson [18]: 0, no deficit; 1, forelimb flexion; 2, as for 1, plus decreased resistance to lateral push; 3, unidirectional circling; 4, longitudinal spinning; 5, no movement. For the grip test [19], the mouse was placed midway on a string between two supports and rated as follows: 0, falls off; 1, hangs on to string by one or both fore paws; 2, as for 1, and attempts to climb on to string; 3, hangs on to string by one or both fore paws plus one or both hind paws; 4, hangs on to string by fore and hind paws plus tail wrapped around string; 5, escape (to the supports).

At 2 h and 12 h after the induction of tMCAO, subgroups of wild-type mice were randomized to receive either 2 mg of the NADPH oxidase inhibitor VAS2870 [9,10] or carrier solution (10% DMSO, Sigma) intrathecally, as described previously [20]. In another group 100 μg apocynin per mouse was injected intravenously 1 h after the occlusion of the middle cerebral artery. Finally, intrathecal injections of H_2_O_2_ (15 mg/kg) were given to another group of *NOX4^-/-^* mice immediately after the occlusion of the MCA and then every hour until 6 h post stroke induction.

Cortical photothrombosis was induced in 6–8-week-old wild-type or *NOX4*^–/–^ mice weighing 20–25 g as described previously [21,22]. In brief, mice were placed in a stereotactic device. A cold light source was positioned 2 mm posterior and 2.4 mm lateral to the bregma. Rose Bengal solution (0.2 ml; Sigma, St. Louis, MO, USA; 10 g/l in normal saline) was administered intraperitoneally, and the brain was illuminated through the intact skull for 20 min. Animals were kept in inhalation anesthesia (2.5% enflurane in a 70% N_2_O, 30% O_2_ mixture) and normothermia throughout surgery.

## Stroke analysis

To determine the permeability of the cerebral vasculature and brain edema, 2% Evans blue tracer (Sigma Aldrich, Germany) diluted in 0.9% NaCl was injected intravenously 2 h after the induction of tMCAO [23,24]. We then performed planimetric measurements (ImageJ software, National Institutes of Health, USA) on 2 mm-thick sections of the brain parenchyma stained by Evans blue to calculate edema volumes.

## Vital brain slices

To prepare vital brain slices, we deeply anesthetized wild-type mice with isoflurane and decapitated them 24 h after tMCAO. Sham-operated mice served as controls. A coronal block of tissue containing the infarct (between –2 mm and –4 mm from bregma) was removed and transferred into ice-chilled saline containing (mM) sucrose, 200; PIPES, 20; KCl, 2.5; NaH_2_PO_4_, 1.25; MgSO_4_, 10; CaCl_2_, 0.5; and dextrose, 10; and pH adjusted to 7.35 with NaOH. Coronal sections (300 µm) through the infarcted region were prepared on a vibratome (Gala instruments, Bad Schwalbach, Germany). Slices were kept submerged in standard artificial cerebrospinal fluid (ACSF; mM): NaCl, 125; KCl, 2.5; NaH_2_PO_4_, 1.25; NaHCO_3_, 24; MgSO_4_, 2; CaCl_2_, 2; and dextrose, 10; pH was adjusted to 7.35 by bubbling with a mixture of 95% O_2_ and 5% CO_2_. Under these conditions brain slices remain vital for 6–8 h [25,26]. Then, slices were incubated with VAS2870 (10 μM) or carrier solution (1% DMSO) for 30 min, cryoconserved, and stained with dihydroethidium to visualize reactive oxygen species.

## Quantitative PCR analysis

Tissue homogenization, RNA isolation, and real-time RT-PCR were performed as described [27,28]. Total RNA was prepared on day 1 after tMCAO in wild-type mice by using a Miccra D-8 power homogenizer (ART, Germany) using the TRIzol reagent® (Invitrogen, Germany) and was quantified spectrophotometrically. 250 µg of total RNA was reverse transcribed with the TaqMan® reverse transcription reagents (Applied Biosystems, Germany) according to the manufacturer's protocol, using random hexamers. Relative levels of *NOX4* mRNA were quantified by using the fluorescent TaqMan® technology. We used TaqMan® gene expression arrays (PCR primers and probes) specific for murine *NOX4* (Applied Biosystems, Germany; assay ID Mm00479246_m1). 18s rRNA (TaqMan® predeveloped assay reagents; part number 4319413E, Applied Biosystems, Germany) was used as an endogenous control to normalize the amount of sample RNA. PCR was performed with equal amounts of cDNA in the GeneAmp 7700 sequence detection system (Applied Biosystems, Germany) using the TaqMan® universal PCR master mix (Applied Biosystems, Germany). Reactions (total volume 50 μl) were incubated at 50˚C for 2 min, at 95˚C for 10 min followed by 40 cycles of 15 s at 95˚C and 1 min at 60˚C. Water controls were included to ensure specificity. Each sample was measured in triplicate. Data points were examined for integrity by analysis of the amplification plot. The comparative Ct method was used for relative quantification of gene expression as described previously [27,28].

## Histology and immunohistochemistry

For immunohistochemical detection of NOX4, tissue was permeabilized by adding 0.2% Triton X-100 in PBS for 1 min. Nonspecific binding was prevented by blocking with 5% goat serum in 1% BSA/GB (gold buffer: 10 mM Tris,
155 mM NaCl, 2 mM EGTA, 2 mM MgCl_2_; pH 7.2) for 20 min. A NOX4-specific primary antibody [12] against human and mouse NOX4 was applied at a dilution of 1:200 in 1% BSA/GB overnight at 4°C. Subsequently, the slides were incubated with a secondary antibody linked to Cy3 (donkey anti-rabbit Cy3, Dianova # 711-165-152) diluted 1:100 in 1% BSA/GB for 1 h at room temperature. Controls included omission of primary or secondary antibodies and gave negative results. In double labeling experiments we used the neuronal marker NeuN (mouse neuN MAB377, clone A60; Millipore, Billerica, USA; 1:1,000) or the endothelial marker von Willebrand factor (VWF; Dako, Germany; monoclonal mouse anti-human vWF, clone F8/86, code NR; M616, lot 094; 1:25) and secondary goat-anti-mouse antibody (Invitrogen, Germany; Alexa fluor 488, 1:300, A11001).

## Oxidative stress markers

Grayscale analysis was performed on inverted digital images by using Scion Image (Version 4.0.3.2, for Win95/98 and Windows NT, Scion Corporation), and the number of reactive oxygen species positive cells per mm^2^ (n = 4 per group) was quantified.

Immunohistochemical staining for nitrotyrosine was conducted on frozen sections taken from identical regions of the mouse brain (–0.5 mm from bregma) 12 h and 24 h after tMCAO. A rabbit polyclonal antibody to nitrotyrosine (Upstate Schwalbach, Germany, Cat. 06-284) was applied at a final dilution of 1:150. Sections were incubated at RT for 2 h, washed three times for 10 min in 1 x PBS and incubated at RT for 45 min with Alexa594-conjugated goat anti-rabbit secondary antibody (1:600, Molecular Probes, Eugene, Oregon, USA; Cat. A-11007). For quantification, we performed grayscale analysis of digital images by using Scion Image (Version 4.0.3.2, for Win95/98 and Windows NT, Scion Corporation), and counted the number of nitrotyrosine-positive cells per mm^2^ (n = 4 per group).

Apoptotic neurons were visualized by TUNEL. Paraffin-wax embedded slices were de-waxed for 30 min in xylol and rehydrated in ethanol. The TUNEL *in situ* death detection kit TMR red (Roche, Switzerland) was used according to the manufacturer’s instructions. Afterwards, slices were washed and subsequently covered with AquaTec (Merck, Darmstadt, Germany). Pictures were collected by immunofluorescence microscopy (Axiophot; Zeiss, Jena, Germany). Quantification of TUNEL-positive cells per mm^2^ (*n* = 4 per group) was performed by using ImageJ software (<http://rsb.info.nih.gov/ij/>). NeuN immunolabeling and TUNEL were performed together on cryopreserved brain slices. Slices were thawed for 30 min and thereafter fixed in acetone at –20°C for 10 min, dried for 10 min at RT, and blocked with blocking solution (10 mM PBS, 5% BSA, 1% goat serum, 0.3% Triton X-100) for 1 h. Primary antibody (mouse neuN MAB377, clone A60; Millipore, Billerica, USA) was diluted 1:1,000 in 10 mM PBS containing 5% BSA and 1% goat serum and incubated at 4°C overnight. After three washing steps (5 min in 10 mM PBS), secondary antibodies (Alexa 488-labeled goat anti-mouse; Invitrogen, Karlsruhe; 1:100) were incubated for 1 h at RT. After washing for 5 min slices were subjected to TUNEL, as described above. Finally, slices were washed and covered with AquaTec (Merck, Darmstadt, Germany).

Visualization was done by using a Zeiss Axiovert 200 M microscope, a CCD camera (Intas, Goettingen, Germany), and Image-Pro® MDA software version 5.1.2.59 (Media Cybernetics, Inc., Bethesda, USA).

## Quantification of protein expression by Western analysis

Homogenized brain tissue (40 mg) from either the cortex or the basal ganglia was separated on a 10% SDS-PAGE gel and transferred to a PVDF membrane. After blocking for 1 hour (3% skimmed milk in TBS with 0.5% Tween), membranes were incubated overnight at 4°C with antibody to NOX1 antibody (1:10,000 dilution), NOX2 (1:10,000 dilution), NOX4 (1:2,000 dilution), or GAPDH (1:10,000 dilution) in the blocking solution described above. Membranes were incubated with secondary HRP-labeled goat anti-rabbit antibodies (for NOX1, NOX2, and NOX4) or HRP-labeled goat anti-mouse antibodies (for GAPDH) for 1 h at room temperature. Proteins were visualized by using Luminol-based chemiluminescence (ECL Western blot detection reagent; GE Healthcare, Buckinghamshire, UK) developed by using an AGFA CP1000 developer (Agfa, Mortsel, Belgium). Bands were quantified by densitometric analysis using Quantity One imaging software (Bio-Rad, Hercules, CA, USA) with a Bio-Rad Gel-Doc imaging system, and normalized to the GAPDH band, which served as a loading control.

### Determination of MAP and HR

Mice were maintained on a 12 h day and night cycle with free access to food and water. 14-week-old male wild-type (*n* = 10) and *NOX4*^–/–^ (*n* = 14) mice were anesthetized (isoflurane 2–4%, O_2_) and a radiotelemetry transmitter (TA11PA-C10, Data Sciences International) implanted into the aorta via the carotid artery. After 10 days’ recovery, continuous recordings of diastolic pressure, systolic pressure, and heart rate (HR) were collected over 3 days. Diastolic and systolic pressures and HR were recorded as 10 second averages every 10 min, and MAP was calculated from diastolic and systolic pressures. Data were analyzed as 1 h and 24 h averages and are represented as mean ± standard deviation.

### Hemodynamic measurements in isolated, perfused, and ventilated lungs and in anesthetized mice

Lungs were removed from the chest during deep anesthesia, artificially ventilated, and perfused blood-free as described previously [29,30]. Briefly, after an initial steady-state period, lungs were ventilated for 10 min with a hypoxic gas mixture containing 1.0% O_2_, 5.3% CO_2_, balanced with N_2_, followed by a 15 min period of normoxic ventilation (21.0% O_2_, 5.3% CO_2_, balanced with N_2_). Subsequently, a second hypoxic ventilation maneuver was performed (10 min, 1.0% O_2_). Normoxic pulmonary arterial pressure (PAP) as well as the PAP increase (ΔPAP) upon hypoxic ventilation (= hypoxic pulmonary vasoconstriction) was quantified. ΔPAP values of the second hypoxic challenge were used to calculate the hypoxic vasoconstrictor response. PAP values directly reflect pulmonary vascular resistance, as the lungs were perfused at constant flow. Right ventricular systolic pressure (RVSP) was assessed in anesthetized mice as described previously.

### Assessment of renal function

At week 17, animals were housed for 24 h in metabolic cages for measurement of food and water consumption and collection of urine for the subsequent measurement of urinary excretion of albumin. The urinary albumin excretion rate (AER) was measured by using a mouse albumin ELISA quantification kit (Bethyl Laboratories, Montgomery, TX, USA) as previously described [31].

**Supplementary References**

1. Schneider I, Tirsch WS, Faus-Kessler T, Becker L, Kling E, et al. (2006) Systematic, standardized and comprehensive neurological phenotyping of inbred mice strains in the German Mouse Clinic. J Neurosci Methods 157: 82-90.

2. Gailus-Durner V, Fuchs H, Adler T, Aguilar Pimentel A, Becker L, et al. (2009) Systemic first-line phenotyping. Methods Mol Biol 530: 463-509.

3. Barone FC, Knudsen DJ, Nelson AH, Feuerstein GZ, Willette RN (1993) Mouse strain differences in susceptibility to cerebral ischemia are related to cerebral vascular anatomy. J Cereb Blood Flow Metab 13: 683-692.

4. Connolly ES, Jr., Winfree CJ, Stern DM, Solomon RA, Pinsky DJ (1996) Procedural and strain-related variables significantly affect outcome in a murine model of focal cerebral ischemia. Neurosurgery 38: 523-531; discussion 532.

5. O'Collins VE, Macleod MR, Donnan GA, Horky LL, van der Worp BH, et al. (2006) 1,026 Experimental treatments in acute stroke. Annals of Neurology 59: 467-477.

6. Crossley NA, Sena E, Goehler J, Horn J, van der Worp B, et al. (2008) Empirical evidence of bias in the design of experimental stroke studies: a metaepidemiologic approach. Stroke 39: 929-934.

7. van der Worp HB, de Haan P, Morrema E, Kalkman CJ (2005) Methodological quality of animal studies on neuroprotection in focal cerebral ischaemia. J Neurol 252: 1108-1114.

8. Dirnagl U (2006) Bench to bedside: the quest for quality in experimental stroke research. J Cereb Blood Flow Metab 26: 1465-1478.

9. ten Freyhaus H, Huntgeburth M, Wingler K, Schnitker J, Baumer AT, et al. (2006) Novel Nox inhibitor VAS2870 attenuates PDGF-dependent smooth muscle cell chemotaxis, but not proliferation. Cardiovasc Res 71: 331-341.

10. Stielow C, Catar RA, Muller G, Wingler K, Scheurer P, et al. (2006) Novel Nox inhibitor of oxLDL-induced reactive oxygen species formation in human endothelial cells. Biochem Biophys Res Commun 344: 200-205.

11. Lange S, Heger J, Euler G, Wartenberg M, Piper HM, et al. (2009) Platelet-derived growth factor BB stimulates vasculogenesis of embryonic stem cell-derived endothelial cells by calcium-mediated generation of reactive oxygen species. Cardiovasc Res 81: 159-168.

12. Anilkumar N, Weber R, Zhang M, Brewer A, Shah AM (2008) Nox4 and nox2 NADPH oxidases mediate distinct cellular redox signaling responses to agonist stimulation. Arterioscler Thromb Vasc Biol 28: 1347-1354.

13. Dikalov SI, Dikalova AE, Bikineyeva AT, Schmidt HH, Harrison DG, et al. (2008) Distinct roles of Nox1 and Nox4 in basal and angiotensin II-stimulated superoxide and hydrogen peroxide production. Free Radic Biol Med 45: 1340-1351.

14. Dikalova A, Clempus R, Lassegue B, Cheng G, McCoy J, et al. (2005) Nox1 overexpression potentiates angiotensin II-induced hypertension and vascular smooth muscle hypertrophy in transgenic mice. Circulation 112: 2668-2676.

15. Goyal P, Weissmann N, Grimminger F, Hegel C, Bader L, et al. (2004) Upregulation of NAD(P)H oxidase 1 in hypoxia activates hypoxia-inducible factor 1 via increase in reactive oxygen species. Free Radic Biol Med 36: 1279-1288.

16. Wingler K, Wunsch S, Kreutz R, Rothermund L, Paul M, et al. (2001) Upregulation of the vascular NAD(P)H-oxidase isoforms Nox1 and Nox4 by the renin-angiotensin system in vitro and in vivo. Free Radic Biol Med 31: 1456-1464.

17. Pham M, Kleinschnitz C, Helluy X, Bartsch AJ, Austinat M, et al. (2010) Enhanced cortical reperfusion protects coagulation factor XII-deficient mice from ischemic stroke as revealed by high-field MRI. Neuroimage 49: 2907-2914.

18. Bederson JB, Pitts LH, Tsuji M, Nishimura MC, Davis RL, et al. (1986) Rat middle cerebral artery occlusion: evaluation of the model and development of a neurologic examination. Stroke 17: 472-476.

19. Moran PM, Higgins LS, Cordell B, Moser PC (1995) Age-related learning deficits in transgenic mice expressing the 751-amino acid isoform of human beta-amyloid precursor protein. Proc Natl Acad Sci U S A 92: 5341-5345.

20. Wu WP, Xu XJ, Hao JX (2004) Chronic lumbar catheterization of the spinal subarachnoid space in mice. J Neurosci Methods 133: 65-69.

21. Kleinschnitz C, Braeuninger S, Pham M, Austinat M, Nolte I, et al. (2008) Blocking of platelets or intrinsic coagulation pathway-driven thrombosis does not prevent cerebral infarctions induced by photothrombosis. Stroke 39: 1262-1268.

22. Schroeter M, Jander S, Stoll G (2002) Non-invasive induction of focal cerebral ischemia in mice by photothrombosis of cortical microvessels: characterization of inflammatory responses. J Neurosci Methods 117: 43-49.

23. Austinat M, Braeuninger S, Pesquero JB, Brede M, Bader M, et al. (2009) Blockade of bradykinin receptor B1 but not bradykinin receptor B2 provides protection from cerebral infarction and brain edema. Stroke 40: 285-293.

24. Belayev L, Busto R, Zhao W, Ginsberg MD (1996) Quantitative evaluation of blood-brain barrier permeability following middle cerebral artery occlusion in rats. Brain Res 739: 88-96.

25. Meuth SG, Budde T, Kanyshkova T, Broicher T, Munsch T, et al. (2003) Contribution of TWIK-related acid-sensitive K+ channel 1 (TASK1) and TASK3 channels to the control of activity modes in thalamocortical neurons. J Neurosci 23: 6460-6469.

26. Meuth SG, Kleinschnitz C, Broicher T, Austinat M, Braeuninger S, et al. (2009) The neuroprotective impact of the leak potassium channel TASK1 on stroke development in mice. Neurobiol Dis 33: 1-11.

27. Kleinschnitz C, Hofstetter HH, Meuth SG, Braeuninger S, Sommer C, et al. (2006) T cell infiltration after chronic constriction injury of mouse sciatic nerve is associated with interleukin-17 expression. Exp Neurol 200: 480-485.

28. Kleinschnitz C, Brinkhoff J, Zelenka M, Sommer C, Stoll G (2004) The extent of cytokine induction in peripheral nerve lesions depends on the mode of injury and NMDA receptor signaling. J Neuroimmunol 149: 77-83.

29. Schermuly RT, Dony E, Ghofrani HA, Pullamsetti S, Savai R, et al. (2005) Reversal of experimental pulmonary hypertension by PDGF inhibition. J Clin Invest 115: 2811-2821.

30. Weissmann N, Dietrich A, Fuchs B, Kalwa H, Ay M, et al. (2006) Classical transient receptor potential channel 6 (TRPC6) is essential for hypoxic pulmonary vasoconstriction and alveolar gas exchange. Proc Natl Acad Sci U S A 103: 19093-19098.

31. Lassila M, Seah KK, Allen TJ, Thallas V, Thomas MC, et al. (2004) Accelerated nephropathy in diabetic apolipoprotein e-knockout mouse: role of advanced glycation end products. J Am Soc Nephrol 15: 2125-2138.
